# Comparative genomics of emerging pathogens in the *Candida glabrata* clade

**DOI:** 10.1186/1471-2164-14-623

**Published:** 2013-09-14

**Authors:** Toni Gabaldón, Tiphaine Martin, Marina Marcet-Houben, Pascal Durrens, Monique Bolotin-Fukuhara, Olivier Lespinet, Sylvie Arnaise, Stéphanie Boisnard, Gabriela Aguileta, Ralitsa Atanasova, Christiane Bouchier, Arnaud Couloux, Sophie Creno, Jose Almeida Cruz, Hugo Devillers, Adela Enache-Angoulvant, Juliette Guitard, Laure Jaouen, Laurence Ma, Christian Marck, Cécile Neuvéglise, Eric Pelletier, Amélie Pinard, Julie Poulain, Julien Recoquillay, Eric Westhof, Patrick Wincker, Bernard Dujon, Christophe Hennequin, Cécile Fairhead

**Affiliations:** 1 Bioinformatics and Genomics Programme, Centre for Genomic Regulation (CRG) and UPF, Doctor Aiguader, 88, Barcelona, 08003, Spain; 2 Université de Bordeaux 1, LaBRI, INRIA Bordeaux Sud-Ouest (MAGNOME), Talence, F-33405, France; 3 Institut de Génétique et Microbiologie, UMR8621 CNRS-Université Paris Sud, Bât 400, UFR des Sciences, Orsay Cedex, F 91405, France; 4 APHP, Hôpital St Antoine, Service de Parasitologie-Mycologie, and UMR S945, Inserm, Université P. M. Curie, Paris, France; 5 Département Génomes et Génétique, Institut Pasteur, Plate-forme Génomique, rue du Dr. Roux, Paris, F-75015, France; 6 CEA, IG, DSV, Genoscope, 2 rue Gaston Crémieux, Evry Cedex, 91057, France; 7 Architecture et Réactivité de l‘ARN, Institut de Biologie Moléculaire et Cellulaire du CNRS, Université de Strasbourg, Strasbourg Cedex, F-67084, France; 8 Institut de biologie et technologies de Saclay (iBiTec-S), Gif-sur-Yvette cedex, 91191, France; 9 INRA, UMR 1319 Micalis, Thiverval-Grignon, F-78850, France; 10 Institut Pasteur, Unité de Génétique moléculaires des levures, UMR3525 CNRS, UFR927, Université P. M. Curie, 25 rue du Docteur Roux, Paris Cedex15, F75724, France; 11 APHP, Hôpital Bicêtre, Service de Microbiologie, Paris, France; 12 Present adress: Champalimaud Foundation, Av. Brasília, Lisboa, 1400-038, Portugal; 13 Comparative Genomics Group, CRG-Centre for Genomic Regulation, Doctor Aiguader, 88, Barcelona, 08003, Spain

**Keywords:** *Candida glabrata*, Fungal pathogens, *Nakaseomyces*, Yeast genomes, Yeast evolution

## Abstract

**Background:**

*Candida glabrata* follows *C. albicans* as the second or third most prevalent cause of candidemia worldwide. These two pathogenic yeasts are distantly related, *C. glabrata* being part of the *Nakaseomyces*, a group more closely related to *Saccharomyces cerevisiae.* Although *C. glabrata* was thought to be the only pathogenic *Nakaseomyces*, two new pathogens have recently been described within this group: *C. nivariensis* and *C. bracarensis*. To gain insight into the genomic changes underlying the emergence of virulence, we sequenced the genomes of these two, and three other non-pathogenic *Nakaseomyces*, and compared them to other sequenced yeasts*.*

**Results:**

Our results indicate that the two new pathogens are more closely related to the non-pathogenic *N. delphensis* than to *C. glabrata.* We uncover duplications and accelerated evolution that specifically affected genes in the lineage preceding the group containing *N. delphensis* and the three pathogens, which may provide clues to the higher propensity of this group to infect humans. Finally, the number of Epa-like adhesins is specifically enriched in the pathogens, particularly in *C. glabrata*.

**Conclusions:**

Remarkably, some features thought to be the result of adaptation of *C. glabrata* to a pathogenic lifestyle*,* are present throughout the *Nakaseomyces,* indicating these are rather ancient adaptations to other environments. Phylogeny suggests that human pathogenesis evolved several times, independently within the clade. The expansion of the *EPA* gene family in pathogens establishes an evolutionary link between adhesion and virulence phenotypes. Our analyses thus shed light onto the relationships between virulence and the recent genomic changes that occurred within the *Nakaseomyces*.

**Sequence Accession Numbers:**

*Nakaseomyces delphensis*: CAPT01000001 to CAPT01000179

*Candida bracarensis*: CAPU01000001 to CAPU01000251

*Candida nivariensis*: CAPV01000001 to CAPV01000123

*Candida castellii*: CAPW01000001 to CAPW01000101

*Nakaseomyces bacillisporus*: CAPX01000001 to CAPX01000186

## Background

Opportunistic fungal pathogens have become a major source of life-threatening nosocomial infections. This situation is partly explained by modern medical progress, relying on large-spectrum antibiotics, immunosuppressive chemotherapy, and devices such as catheters, all of which have been shown to predispose to invasive candidiasis [1].

Among the emerging fungal pathogens, the incidence of *Candida glabrata* has progressively increased, and it is currently the second or third most prevalent cause of candidiases. Despite its name, this yeast is phylogenetically closer to the model yeast *Saccharomyces cerevisiae* than to *C. albicans*[2], and is part of the *Nakaseomyces* genus. This genus originally included three other species of yeasts isolated only from the environment, namely *Nakaseomyces* (*Kluyveromyces*) *delphensis*, *Candida castellii* and *Nakaseomyces* (*Kluyveromyces*) *bacillisporus*[3]. Recently however, two pathogens have been added to the genus, *Candida nivariensis* and *Candida bracarensis*[4,5]. Because routine phenotypic tests, such as biochemical identification methods, are unable to identify these newly described species, leading most often to misidentification as *Zygosaccharomyces* (CH and AEA, unpublished) their true clinical relevance may be underestimated. Recently, collections of clinical isolates, phenotypically identified as *C. glabrata,* were screened with molecular methods, and *C. bracarensis* and *C. nivariensis* were found to represent less than 2.2% and less than 0.1% of the strains, respectively, with prevalence possibly varying across countries [6,7]. Interestingly, although *C. glabrata* is considered a commensal of the human gut [8], the ecological niches of *C. bracarensis* and *C. nivariensis* remain unknown. Of note, *C. nivariensis* has been isolated from flowering plants in Australia [9], pointing to the possibility that this species may colonize humans from an environmental source.

As it is often the case in fungi, loss, or scarcity of sexual reproduction is associated to species isolated in human patients. Nonetheless, the *Nakaseomyces* comprise at least one known “environmental” species in which no sex has been observed, *C. castellii*[10]. *MAT*-like loci and the *HO* gene, the key player of mating-type switching in *S. cerevisiae*[11], were known to be conserved in *C. glabrata* and *N. delphensis*[12].

The genomic sequence of *C*. *glabrata* has been available since 2004 [2], and its comparison to *S. cerevisiae* has served to discuss possible genomic and metabolic features related to the pathogenic nature of the former [13]. However, it was as yet unclear whether some of these features were also shared by other *Nakaseomyces* and how their presence actually related to the ability of the different species to become human pathogens. In the case of *Candida albicans*, this has been explored by sequencing several of its close relatives [14]. To gain a similar insight into the specific features of *C. glabrata* and their relation to pathogenicity, we now report the complete sequencing of the five other known species in the *Nakaseomyces* group.

Our results show that all *Nakaseomyces* nuclear genomes are small, transposon-free and contain significantly less genes than *S. cerevisiae*. This is in contrast to their mitochondrial genomes which, with the exception of *C. glabrata*, are large and invaded by palindromic putative mobile elements, the GC inserts [15]. Loss of genes involved in several metabolic pathways as well as loss or amplification of some gene families, are shared by most, sometimes all, *Nakaseomyces* species, although some remain species-specific. Our molecular phylogenetic analysis supports the phylogeny of the *Nakaseomyces*, as published by Kurtzman, [3] ie all these species can be grouped together as a new genus with a single common ancestor. We also confirm that the genus can be clearly subdivided into two main lineages, where the lineage containing *C. castellii* and *N. bacillisporus* has followed a very divergent evolutionary path. The second group, which we will refer to as the ‘*glabrata* group’, contains the three pathogenic species and *N. delphensis*, which is more closely related to the two recently identified pathogens. This depicts a complex scenario suggesting multiple independent events of emergence of pathogenesis within this lineage, and the presence of a genomic repertoire that may facilitate the emergence of pathogenicity towards humans. Altogether our comparative analyses have enabled us to trace, at high levels of resolution, the genomic changes that occurred within this group and discuss how they relate to the pathogenic ability of the different species.

## Results

### Genome assemblies

Sequencing of the type strains of the five *Nakaseomyces* species; *Candida nivariensis* CBS9983, *Candida bracarensis* CBS10154, *Nakaseomyces* (*Kluyveromyces*) *delphensis* CBS2170, *Candida castellii* CBS4332, and *Nakaseomyces* (*Kluyveromyces*) *bacillisporus* CBS7720, was performed at Genoscope (Evry, France), using a combination of Illumina and 454 technologies (see Methods). Genome data has been deposited at the EMBL.

Final assemblies showed a close correspondence between scaffolds and chromosomes (Additional file 1), and were annotated for coding and non-coding genes (see Methods). Flow cytometry results show that species are haploid, except *N. bacillisporus* which is diploid (Additional file 2). All species have an haploid genome size of 10 to 12 Mb. Chromosome numbers, as estimated by Pulsed-Field Gel Electrophoresis (not shown), range from eight in *C. castellii,* the smallest yet recorded number of chromosomes in post-WGD yeasts [16], to fifteen in *N. bacillisporus*; with the ‘*glabrata* group’ exhibiting the least variation (10–13 chromosomes). Centromeres, similar in structure to *S. cerevisiae*’s, i.e. composed of three short “centromere defining elements”, CDEI, II and III [17]; were identified in all species but not in all scaffolds. Telomeric repeats identical to those in *C. glabrata* and the putative telomerase RNA-component were found in the *‘glabrata* group’.

Several ncRNA genes are known to be surprisingly large in *C. glabrata*, such as the *RPR1* gene of RNAse P [18] and the above-mentioned *TLC1* gene [19]. This tendency to exceptionally large ncRNAs seems to be general in the *Nakaseomyces*, such as the 1368 nt-long *RPR1* gene in *N. delphensis* (only 369 nt-long in *S. cerevisiae)*, and the 1937 nt-long U1 snRNA gene in *C. castellii* (568 nt-long in *S. cerevisiae*). Other structural genomic features are discussed in the supplementary results (see Additional file 3). None of the species contain detectable active transposons in their nuclear genome.

### Phylogeny of *Nakaseomyces*

**Figure 1:**
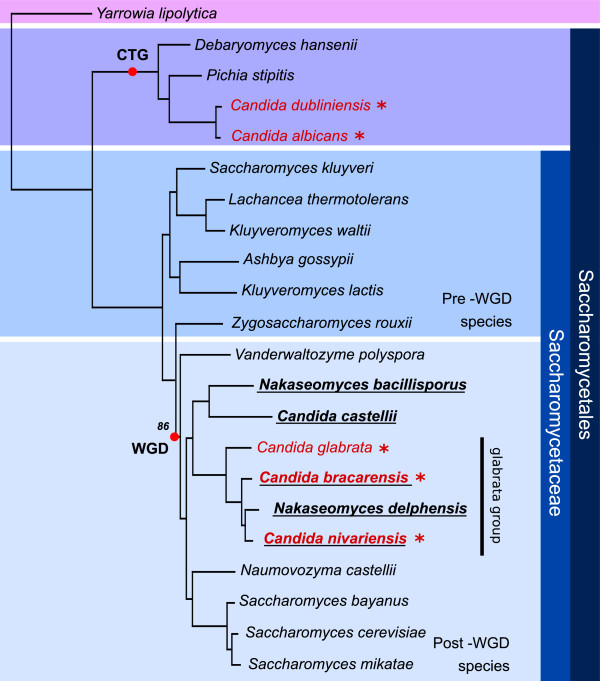
**Maximum likelihood species tree of 22 *Saccharomycotina *species.** The tree was reconstructed based on the analysis of a concatenated alignment of one-to-one orthologs of 603 widespread genes. Species names in red and with an asterisk indicate human fungal pathogens. Underlined species names correspond to the newly sequenced *Nakaseomyces* species. Important evolutionary events such as the Whole Genome Duplication (WGD) or the genetic code transition in the *Candida* clade (CTG) are marked on the tree. All aLRT-based supports were maximal and a single node with a bootstrap support below 100% is indicated. This topology is also the most parsimonious in terms of inferred duplications in 4,965 individual gene phylogenies, as assessed by a Gene Tree Parsimony approach implemented in duptree [20].

In order to clarify the phylogenetic relationships of the newly-sequenced *Nakaseomyces* and 17 other *Saccharomycotina*, we used two alternative phylogenomics approaches, namely i) a Maximum Likelihood (ML) analysis of a concatenated alignment of 603 protein families that have one-to-one orthologs in all the species considered, and ii) a super-tree approach based on the analysis of 4,965 individual gene trees, which finds the species topology that is most parsimonious in terms of implied duplication events in all the individual gene trees [20]. Both approaches yielded the same, highly resolved topology (Figure 1), which is largely congruent, for the shared species, with our current understanding of *Saccharomycotina* phylogeny [21].

This topology supports the the existence of the *Nakaseomyces* genus, and defines two clear sub-groups separated by an ancient split. The first group comprises the highly divergent *C. castellii* and *N. bacillisporus*, whereas the second group (i.e. the ‘*glabrata* group’) includes the three pathogenic species and *N. delphensis*. The average protein identity between orthologs of species in the different sub-groups ranges from 51 to 53% (Additional file 4), which is similar to that of orthologs in the *C. castellii/N. bacillisporus* (53%) and *C.glabrata/S.cerevisiae* (54%) pairs, pointing to large levels of divergence. The *‘glabrata* group’, is more compact and shows higher identity levels (77-88%). Notably, the two newly identified pathogens, *C. nivariensis* and *C. bracarensis*, are both closer to *N. delphensis* than to the most frequently isolated pathogen, *C. glabrata* (Figure 1). Thus the emerging picture for the appearance of pathogenesis is complex, with plausible alternative scenarios involving either gain of the ability to infect humans at the base of the sub-clade followed by loss of the trait in *N. delphensis* or three independent events of acquisition of pathogenicity. These possibilities will be further discussed below.

The degree of synteny, i.e. gene order conservation between species, was found to correlate with phylogenetic proximity, with the highest conservation occurring between *C. bracarensis*, *C. nivariensis* and *N. delphensis* (Additional file 5). Conservation of synteny of the *Nakaseomyces* in general, relative to *S. cerevisiae* is low. Detailed analysis of syntenic regions will be presented elsewhere (HD, TG, CF, in preparation).

### Mating types

As is the case for many fungal species described as asexual, genes involved in sexual reproduction are known to be conserved in *C. glabrata*[22-24]. In particular, although mating has never been reported in *C. glabrata*, the two additional *MAT* cassettes, *HMRa* and *HMLalpha*, and the *HO* gene are present in its genome, and haploid isolates of both mating types are found. In *N. delphensis*, also a mainly haploid species, these elements are also conserved [12,23] and mating-type switch may occur in culture [12]. For the remaining four species, three are considered asexual and mainly haploid, but the last one, *N. bacillisporus*, is considered to be diploid and homothallic [25]. As mentioned above, ploidy was confirmed by flow cytometry. All species contain a well-conserved homolog of the *HO* gene (Additional file 6), the most diverged encoded protein being the one from *N. bacillisporus*, which exhibits a C-terminal extension rich in proline and serine.

**Figure 2:**
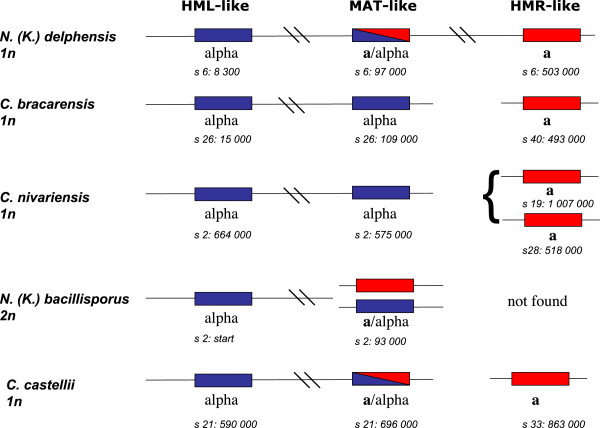
***MAT*-like cassettes in the sequenced genomes.***HMR*-, *HML*- and *MAT*-like are represented as rectangles, with red ones containing **a**-type information and blue ones containing alpha-type information. *MAT* cassettes of each type are shown for *N. bacillisporus*, which is diploid, and bicolored *MAT* cassettes in haploid species indicate possible switching in culture. Approximate coordinates on corresponding scaffold are shown below each cassette.

In all species, additional sequencing was needed in order to obtain the cassettes. In *N. delphensis*, *N. bacillisporus* and *C. castellii*, amplification of the *MAT*-like cassette yielded both a and alpha fragments. This raises the intriguing possibility that *C. castellii* switches mating types in culture, whilst having no described sexual cycle, or that it is sexual and goes through a diploid phase. This will need further experimental analysis. Figure 2 shows which cassettes are currently identified in these genomes. Genes within cassettes, a1, alpha1 and alpha2 are identified in all species, as is the Ho site, which can be recognised at the YZ junction. In cases where the three cassettes are identified, their configuration is apparently similar to *C. glabrata*’s: both *HML-* and *MAT*-like cassettes are on the same scaffold, while the *HMR*-like cassette is on a different one, except for *N*. *delphensis*, where the three cassettes are on the same scaffold. Thus, *Nakaseomyces* species follow the rule of conservation of *HML* and *MAT* on the same chromosome, noticed by Gordon et al. [26].

*C. nivariensis* has two *HMR*-like cassettes, i.e. cassettes that contain type **a** information, and that are present in addition to the *MAT* locus, a situation already noted in other species [26]. Experimental testing of these cassettes will reveal what role, if any, these extra loci have, in organisms where sexual reproduction has not been characterized yet.

### Variations in gene repertoires

The total numbers of protein-coding genes range from 5400 to 5900, which is similar to what is found in *C. glabrata*[2], and lower than in *S. cerevisiae*. Indeed, the number of true protein-coding genes in *S. cerevisiae* is estimated at around 5800, but this rises to around 6600 when dubious and other non-experimentally characterized ORFs are included, a figure more comparable to our predicted gene sets (SGD, 12 January 2012, http://www.yeastgenome.org, and Additional file 7). To assess the coverage of the predicted gene repertoires we tested for the presence of a set of 2,007 protein families previously found to be widespread in *Saccharomycotina*[21]. Our proteomes contained 99.4-100% of this core dataset, attesting for a high coverage in our procedures.

Intron-containing protein-coding genes are far fewer in *C. glabrata* than in *S. cerevisiae* (129 *vs* 287, [27]). This paucity of introns is shared by all *Nakaseomyces*, which have intron counts lower than 230. Although experimental validation is needed, our data point to a remarkable loss of introns in the *Nakaseomyces*.

**Figure 3:**
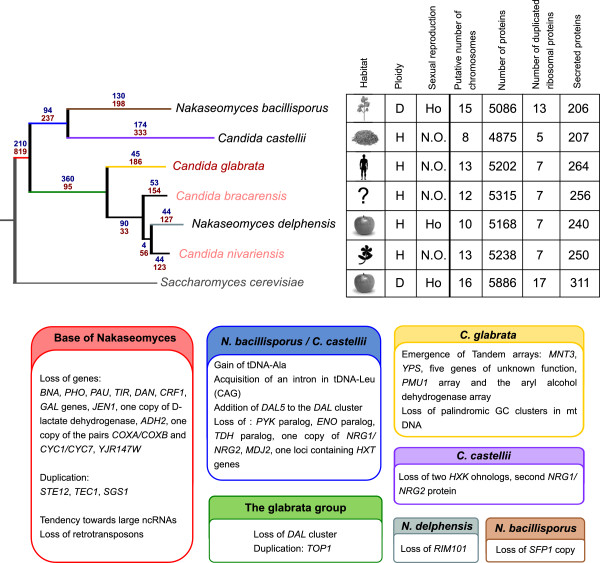
**Summary of the main findings.** The phylogenetic tree represents the evolution of the *Nakaseomyces* species, using *S. cerevisiae* as outgroup. Blue numbers indicate the number of genes that are predicted to have been gained at each lineage during the evolution of the *Nakaseomyces*. Red numbers indicate the yeast genes that have been lost. Species names coloured in red indicate the human pathogens, lighter colouring indicates recently-reported emerging pathogens. Coloured branches can be matched to the corresponding coloured boxes below, which list important events occurring at that lineage in the evolutionary history of *Nakaseomyces*. (Abbreviations: Ploidy: H: Haplobiontic, D: Diplobiontic; Sexual reproduction: H: homothallic, N.O.: not observed).

**Figure 4:**
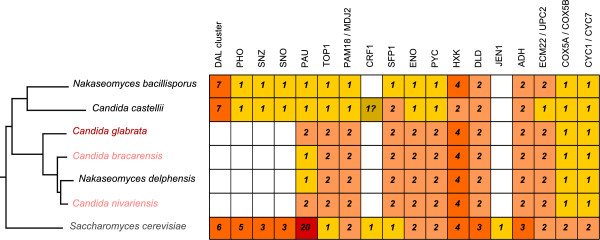
**Phylogenetic profiles of specific gene families and pathways.** The phylogenetic tree represents the evolution of the *Nakaseomyces* species, with the pathogens colored. White boxes indicate absence of a particular family or pathway in a given species, while numbers in colored boxes indicate the number of paraloguous copies of that gene family or the number of components of a given pathway. Intensity of the colors is proportional to the number of paralogs present.

To accurately trace the evolution of the genetic repertoires across the *Nakaseomyces*, and confidently establish orthology and paralogy relationships, we performed an exhaustive phylogenomic analysis that included the reconstruction of ML phylogenies for every gene encoded in the *Nakaseomyces* genomes (i.e. the phylome). These were used to detect orthology and paralogy relationships [28], and to map lineage-specific gene duplication [29] and gene loss events onto the species tree. Figures 3 and 4 and Additional file 8 summarize the main findings regarding the presence or absence of genes relevant to central processes. Lower gene numbers in the *Nakaseomyces* when compared to *S. cerevisiae* are reflected in the number of gene loss events indicated on the tree from Figure 3.

In particular, we have paid special attention to the retained copies of so-called ohnologs (i.e. gene duplicate pairs originated during the WGD event; [30]). In *S. cerevisiae*, only 551 ohnologous pairs are left in the contemporary genome, as most such pairs have lost one member [31]. This is also the case in the *Nakaseomyces* genomes, as in all post-WGD species. The exact complement of ohnologs retained in duplicates varies across species and is likely to reflect particular physiological differences.

A noteworthy example comes from central carbon metabolism: it has been suggested that the six ohnologous gene pairs encoding glycolytic enzymes, such as *ENO1*/*ENO2*, and *PYC1*/*PYC2*, conserved in *S. cerevisiae* and *C. glabrata*, play an important role in the Crabtree effect, ie fermentation even in the presence of high glucose and oxygen [32], a trait shared by all *Nakaseomyces*[33,34]. The genes involved are also in pairs in the ‘*glabrata* group’, but, in both *C. castellii* and *N. bacillisporus*, the situation is rather different, with single pyruvate kinase, enolase, and Glyceraldehyde-3-phosphate dehydrogenase genes (Figure 4, and Additional file 9). Furthermore, *C. castellii* has two hexokinase genes (*HXK*), as compared to four (two pairs of ohnologs) in the other species. Other features of central carbon metabolism in the *Nakaseomyces* are mentioned below and in Additional file 3.

Comparison of the proteins encoded in *C. glabrata* and *S. cerevisiae*’s genomes had revealed several features specific to the former. In particular, since there are fewer genes in *C. glabrata* than in *S. cerevisiae*, there was a special interest in specific gene loss. Indeed, there are at least four entire multigenic families which are absent in all *Nakaseomyces* or represented by a sole member in *C. castellii* and/or *N. bacillisporus*: the *PHO*, *SNZ*, *SNO* and *PAU* families. It is noteworthy that the paralogous *PHO* family of acid phosphatases has been lost, while the rest of the *PHO* pathway is conserved. Functional analysis in *C. glabrata* has shown that the *PHO2* gene is present but not essential for regulation, and that the *PHO4* gene is poorly conserved at the sequence level, but is functional [35]. Phosphate-starvation induced phosphatase activity in *C. glabrata* has been identified and is encoded by *PMU1* homologs in tandem ([36], and see section on tandem arrays). The *SNZ* and *SNO* gene families are poorly characterized in *S. cerevisiae*, but their expression is known to be induced in stationary phase. Finally, the *PAU* family consists of at least 20 subtelomeric genes in *S. cerevisiae*, possibly involved in anaerobiosis [37] and similar to two other gene families, *DAN* and *TIR*, that encode cell-wall mannoproteins. There are no homologs to any of these genes in *C. castellii* and *N. bacillisporus*, while *C. glabrata* and *C. nivariensis* contain two copies of *DAN*-like genes, and the remaining species seem to contain only one homologous copy.

Also of particular interest was the loss of genes involved in *de novo* biosynthesis of nicotinic acid (*BNA)*, which was hypothesized to result from the adaptation of *C. glabrata* to its human host [38]. Comparison to the newly sequenced *Nakaseomyces* has shown that the lack of *BNA* genes is common to them all (Additional file 8), regardless of their habitat, suggesting that the loss of this pathway is an ancient event. Notably, loss of *BNA* genes has also been observed in other, more distant species, such as *Kluyveromyces lactis* and other species, and seems to be a volatile trait [39]. In fact, all gene losses observed in *C. glabrata* relative to *S. cerevisiae* are shared by the ‘*glabrata* group’, whereas in the two other species gene absence is variable. A remarkable example concerns the genes necessary for allantoin catabolism. In *S. cerevisiae*, the subtelomeric *DAL* (degradation of allantoin) cluster consists of six genes, and was formed in the ancestor to *S. cerevisiae* and *Naumovia castellii*, but lost in *C. glabrata*[40]. The *DAL* cluster is absent from the ‘*glabrata* group’, but is present in both *C. castellii* and *N. bacillisporus*. Intriguingly, in these genomes, the cluster contains another gene involved in allantoin catabolism, *DAL5*, which, in *S. cerevisiae*, is not linked to the cluster (Additional file 10).

Other notable examples of gene gain/loss involve the translation machinery: both the number of ohnologous ribosomal protein (RP) gene pairs and the RP gene regulators, *CRF1* and *SFP1*, vary between species, with no correlation between absence/presence of regulator genes and number of ohnologous RP gene pairs (Figure 4, Additional files 3 and 11).

Central carbon metabolism again provides examples of gene gain/loss events; all *Nakaseomyces* genomes contain only two copies of *ADH* genes, which, both by similarity and by conservation of synteny, correspond to orthologs of *ADH1* and *ADH3*, the *S. cerevisiae* genes that encode, respectively, the cytoplasmic and the mitochondrial activities converting acetaldehyde into ethanol. *ADH2*, which in *S. cerevisiae* is specialized in the conversion of ethanol to acetaldehyde, has no ortholog in the *Nakaseomyces*. It is possible that bi-directional activities exist, or that alternative enzymes take over this conversion (i.e. co-option). For example, *S. cerevisiae* has additional alcohol dehydrogenase genes, in particular the family of aryl *alcohol* dehydrogenases encoded by seven subtelomeric *AAD* genes and the non-subtelomeric *YPL088W* gene [41]. *C. glabrata* possesses an array of three such genes in tandem, while other *Nakaseomyces* have several dispersed copies, except *C. castellii* which harbors a single such gene. Experimental analysis is needed to tell which enzymes catalyze which reactions in *Nakaseomyces*, and even in *S. cerevisiae*, in which enzymatic activities are still in the process of being characterized [42]. As for anaerobic growth, all *Nakaseomyces* have the ability to grow in micro-aerobiosis, as tested by standard laboratory methods (not shown). Two pairs of regulators are described as essential to anaerobiosis in *S. cerevisiae*; the *ECM22*/*UPC2* pair and the *SUT1*/*SUT2* pair. These genes are conserved in all *Nakaseomyces* except *C. castellii*. There are also pairs of genes that differ by their expression under aerobic and anaerobic growth, such as *COX5A*/*COX5B* and of *CYC1*/*CYC7* in *S. cerevisiae*. In contrast, all *Nakaseomyces* retain a single member of each of these ohnologous pairs, raising the question of their regulation.

### Genes involved in virulence

**Figure 5:**
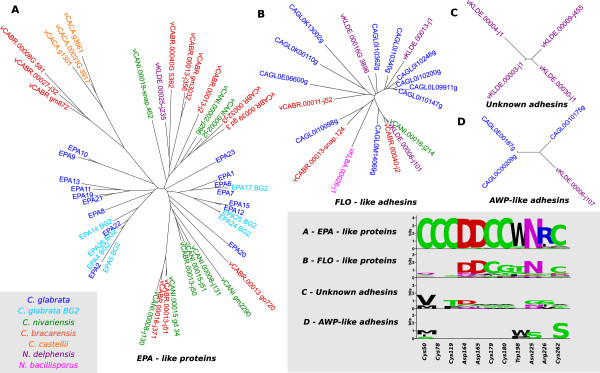
**Trees of the three groups of adhesins containing more than one member.** Phylogenetic trees were reconstructed using the same approach as for the trees present in the phylome, and are visualized using ETE [50], **A)** proteins grouped with the known *C. glabrata* EPA genes, **B)** proteins similar to the yeast Flo protein, **C)** group of unknown adhesins, and **D)** sequences related to AWP adhesins. Labels indicate protein names and are colored depending on the species they belong to: *C. glabrata* in dark blue, a second strain of *C. glabrata* (BG2) in light blue, *C. bracarensis* in red, *C. nivariensis* in green, *N. delphensis* in purple, *N. bacillisporus* in yellow and *C. castellii* in orange. Logos at the lower right part of the image represent the conservation in each of the adhesin groups of sites that are considered important in the structure of *EPA1*[51].

In *C*. *glabrata*, the *EPA* genes, a family of glycosyl-phosphatydylinositol (GPI)-anchored cell-wall protein (CWP) genes [43], are the best characterized genes involved in adhesion to human epithelia [44,45], an ability associated to virulence in diverse pathogens [46]. Notably, our search for homologs of *C. glabrata EPA* genes in the newly sequenced *Nakaseomyces* (see methods), revealed higher numbers of such genes in the three pathogenic species. More specifically, in the most prevalent pathogen, *C. glabrata*, the type strain harbors 18 members of this family. Seven new variants of *EPA* genes are found in a different *C. glabrata* strain, BG2 (data kindly provided by Brendan Cormack). The other two pathogenic *Nakaseomyces*, *C. bracarensis* and *C. nivariensis* have respectively, 12 and 9 members of the *EPA* family. In contrast, the non-pathogenic *N. delphensis* harbors a single copy. These differences cannot be attributed to differences in the quality of assemblies, which were similar in all newly sequenced species. Of the two remaining *Nakaseomyces* species, only *C. castellii* contains three homologs of the *EPA* genes, while *N. bacillisporus* presented only one distant homolog that clustered with PWP (PA-14 containing Wall Protein, adhesin gene) and adhesin-like protein genes in *C. glabrata*. These proteins, more similar to the floculin homologs in *S. cerevisiae*, are only distantly related to Epa proteins [47,48]. A closer inspection of the corresponding trees in the phylome and of a composite tree constructed with all members of the identified *EPA*-like members (Figure 5a), revealed that a significant fraction of the adhesin gene copies in *C. glabrata* emerged from lineage-specific duplications in this species, whereas many duplications in *C. bracarensis* and *C. nivariensis* are shared. This independent expansion of *EPA*-like genes in *C. glabrata* and the emerging pathogens supports the idea of an independent emergence of pathogenesis and may explain the important differences in prevalence across these species. Strikingly, the non-pathogenic member of the ‘*glabrata* group’, *N. delphensis* possesses a sole representative of this family, although the phylogenetic scenario implies that the common ancestor of this species and the two emergent pathogens would have had a higher number of copies. This scenario implies specific losses of *EPA*-like genes in *N. delphensis*, and a possible secondary loss of adhesion capabilities in this species. Interestingly, our search for adhesin-like proteins uncovered the presence of a group of four proteins in *N. delphensis* with poor similarity with the *EPA*-like family (Figure 5c). Whether this group represents highly divergent members -or even pseudogenes- of the *EPA*-like family in *N. delphensis,* remains to be further investigated. In any case, their high levels of sequence divergence, particularly at the N-terminal region known to harbor the ligand-binding domain in Epa [49], suggest they cannot be functionally equivalent.

Other genes shown to be involved in virulence in *C. albicans* and/or *C.glabrata*, such as the phospholipase B gene, or the Super Oxide Dismutase genes (*SOD* genes), exhibit variable presence/absence in the *Nakaseomyces*, with no obvious correlation to pathogenicity of the species.

### Genes in tandem arrays

**Figure 6:**
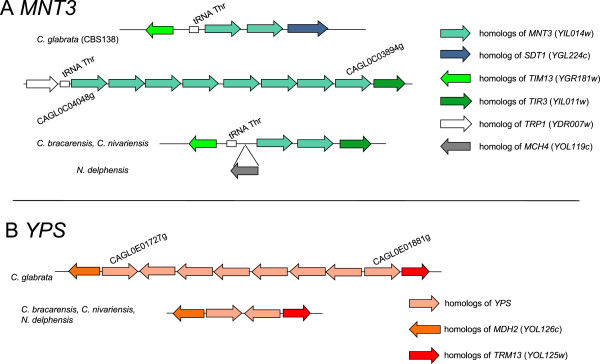
**Tandem repeats of eight genes identified in *C. glabrata *and their homologs in the *‘glabrata *group’. A**: *MNT3* homologs in *C. glabrata* are present in a tandem repeat of eight genes and in a tandem of two genes, with additional isolated copies. Closest species exhibit a tandem repeat of two genes with mixed synteny at the borders. **B**: *YPS* homologs in *C. glabrata* are present in a tandem repeat of eight genes, with additional isolated copies. Closest species exhibit a tandem repeat of two genes with conserved synteny at the borders.

Tandem arrays are a special genomic arrangement of certain gene families that imply specific amplification mechanisms and positive selection. They can either be shared by all members of a species, such as the globin genes in mammals [52], or display polymorphism in terms of number of copies, such as the *CUP1* gene in *S.cerevisiae*, whose amplification is positively selected in copper-containing medium [53]. A few dozen tandems were identified in the *Nakaseomyces* genomes (HD, TG and CF, ms in preparation). The species with the largest tandem arrays is *C. glabrata*, with two arrays of eight genes each (Figure 6), the *MNT3* array (shown to be variable in different strains, [54]) and the *YPS* array [55], and an array of five genes, coding for a protein of unknown function predicted to be involved in carbohydrate metabolism. None correspond to tandem arrays of more than two genes in other *Nakaseomyces*. Two other arrays of three genes are specific to *C. glabrata*, the aryl alcohol dehydrogenase gene array, and the *PMU1* array. These findings indicate that most tandem arrays found in *C. glabrata* originated specifically in this lineage. This, together with the finding that some of these arrays are variable across strains, suggests that the amplification of these families may be driven by (directional) selection. Functions encoded by these families, are thus good candidates for finding possible physiological advantages underlying the success of *C. glabrata* as an opportunistic pathogen. In the case of the *YPS* cluster, the genes have been shown to be involved in virulence [55]. *PMU1* encodes the phosphate-starvation inducible phosphatase activity, which has been hypothesized to be a specific adaptation of *C. glabrata* to its mammalian host [36].

### Analysis of evolutionary rates in the lineages leading to the *‘glabrata* group’

As mentioned above, the ‘*glabrata* group’ contains several species with the ability to infect humans. We have previously discussed changes that occurred within this lineage in terms of acquisition and loss of genes. We next set out to investigate whether we could identify signatures of possible positive selection in the form of genes with accelerated rates in the branch preceding the diversification of the ‘*glabrata* group’. For this we focused on 2,153 genes predicted as one-to-one orthologs shared by all *Nakaseomyces* species and *S. cerevisiae*. Using the species’ phylogeny, we used a likelihood ratio test (LRT) to compare two nested rate models (Additional file 12). For 991 of the 2153 genes (43%), a model including a different rate in the branch leading to the ‘*glabrata* group’ was favored, and in 35 of them there was evidence for accelerated rates, suggestive of positive selection (i.e. *d*_*N*_/*d*_*S*_ rate >1, with an average of 3). In contrast, we did not find a significant rate acceleration in either the *S. cerevisiae*, *N. bacillisporus* or *C. castelli* lineages, nor within the ‘*glabrata* group’ itself, where the average *d*_*N*_/*d*_*S*_ rate was 0.04 indicating high levels of purifying selection. These findings suggest that prior to the diversification of the ‘*glabrata* group’, there was an increase in the non-synonymous substitution rate in at least 35 genes (1.6% of the tested genes). Among these we did not find families that have been related to pathogenesis in *C. albicans.* Finally, we focused on the ‘*glabrata* group’ to identify genes accelerated in parallel in the three lineages leading to the three pathogenic species or exclusively in the *C. glabrata* lineage. We identified 94 proteins with evidence for different selective constrains in pathogenic species and *N. delphensis,* although this difference was mostly due to stronger purifying selection, rather than positive selection, in the pathogens. With respect to the *C. glabrata* specific branch, only four genes presented *d*_*N*_/*d*_*S*_ >1. Overall, these results would suggest a burst of nonsynonymous substitution rates in a significant number of genes preceding the divergence of the *‘glabrata* group’ and a subsequent stasis within the group itself. Nevertheless, genes that are positively selected in these lineages constitute good candidates for testing potential roles in virulence.

## Discussion

Even though experimental data will be needed to clarify the relationship between genome content and adaptation to the environment, we show that many gene variations observed occur in families of genes encoding cell wall proteins (*PAU*, *EPA*, and other ministallite-containing genes), as well as proteins involved in carbohydrate metabolism. These classes of genes have already been shown to be involved in adaptation of yeast species to particular biotechnological niches such as the *MEL* and *MAL* genes in *Saccharomyces* species, [56,57], the *SUL* genes in lager yeasts [58] or in adaptation to the human host such as the *EPA* genes in *C. glabrata*[55] and the *ALS* genes in *C. albicans*[59]. Some of these genes fit the definition of contingency genes, i.e. genes that encode products that mediate the cell’s response to its environment and that evolve faster than allows the average mutation rate of other genes [60]. Rapid evolution is supposed to be facilitated by internal repeats, such as within the *EPA* genes, and by subtelomeric location, situations which enhance recombination frequency and generate new alleles and possibly new genes.

Our data firmly support the existence of the *Nakaseomyces* genus*,* but nonetheless, two of the species, *C. castellii* and *N. bacillisporus*, have diverged in many ways, first by the low conservation of their orthologous genes, when compared to the four other *Nakaseomyces*, and also by their paucity of tandems, and by their frequent variation in gene numbers compared to the ‘*glabrata* group’ (such as in “ohnologous” ribosomal protein gene pairs). Furthermore, our analyses shows that the two emerging pathogens, *C. nivariensis* and *C. bracarensis*, are most closely related to the non-pathogenic *N. delphensis*, and all descend from an ancestor that has also given rise to *C. glabrata*. In accordance to their phylogenetic relationship, most genomic particularities observed in *C. glabrata* as compared to *S. cerevisiae*, are also shared by the two emerging pathogens and *N. delphensis*. Some others, such as the absence of the nicotinic acid synthesis pathway are even common to all *Nakaseomyces*, and thus represent ancient *Nakaseomyces* traits, whose origin must have pre-dated the adaptation to the human host of some of the members of the group. Hence, the scenario for the emergence of pathogenicity within the *Nakaseomyces,* and the underlying genomic and metabolic changes must be reinterpreted in the light of these new genomic data. These findings also highlight the utility of increased taxonomic samplings when correlating genomic and phenotypic differences. Considering that most genomic and metabolic features previously thought to be particular of *C. glabrata* are shared by the three most closely related *Nakaseomyces,* of which two are not natural inhabitants of the human gut, the most plausible explanation is that they result from adaptations to environments other than the human gut. The alternative scenario in which human commensalism is an ancestral trait that was lost in *C. nivariensis* and *N. delphensis* seems unlikely given the large evolutionary distances involved and the relatively recent origin of our species. Furthermore, since these four related species include one that has never been identified as a human pathogen, and two that have only been recently reported as opportunistic pathogens, the link to the emergence of pathogenesis for their specific genomic features should be indirect. Nevertheless, the presence of three related species able to infect humans within this sub-group of *Nakaseomyces* is in stark contrast to the almost complete absence of this ability in the rest of the genus as well as in the related *Saccharomyces* and *Kluyveromyces* groups. Thus, it seems that some of the particularities shared by *C. glabrata* and the three related species may represent pre-adaptations (exaptations in evolutionary terms) that may have facilitated, but not directly triggered, the emergence of pathogenecity towards humans. Although further research is needed to identify which traits may have been particularly important for the emergence of pathogenesis within the *Nakaseomyces*, several of the traits shared by *C. glabrata* and the three closest relatives are firm candidates. These include genes that likely underwent positive selection specifically in this lineage, of which some have homologs implicated in pathogenesis in *C. albicans*. In addition, the multiple parallel expansions of the *EPA* genes, known to be important for the ability of *C. glabrata* to adhere to human cells, represent a clear example of a genomic change that correlates with the pathogenic trait. Our data supports the fact that the emergence of pathogenesis relies on a combination of genomic alterations, rather than changes in a single gene family.

Undoubtedly, *C. glabrata* is the most prevalent pathogen among the *Nakaseomyces.* This increased ability to infect immunocompromised humans is probably related to some of the specific changes observed in the *C. glabrata* lineage, as compared to the other *Nakaseomyces*. These include specific losses, expansions of some gene families, particularly as tandem arrays, and even the acquisition of horizontally transferred genes [61]. Remarkably, some of these traits specific to *C. glabrata* have been related to virulence, such as the largest expansion of gene families involved in cell adhesion (*EPA*) and in phosphate starvation (*PMU1*). That the *EPA* genes have been expanded in *C. glabrata* independently of the emergent pathogens is consistent with the important differences of prevalence and suggest an independent emergence of increased adherence to the human epithelium, an important virulent trait.

Considering all these data, one can speculate on the possible scenarios depicting the origin of pathogenesis within the *Nakaseomyces*. To start with, our phylogeny does not support the monophyly of *C. glabrata* and the two emerging pathogens. Two competing scenarios may explain this pattern: a single origin of pathogenesis towards humans followed by loss of the trait in *N. delphensis* or, alternatively, at least two independent events of emergence of pathogenesis. Although the first scenario is more parsimonious in terms of the number of phenotypic shifts, the second hypothesis seems more likely in the light of the parallel expansions of the *EPA* genes, and the fact that the pathogens other than *C. glabrata* have only been recently identified, suggesting these are recently emerged rather than derived pathogens. A plausible scenario for the emergence of pathogenesis within the *Nakaseomyces*, compatible with our data, comprises the following steps: an ancestral environmental yeast with specific genomic features, gives rise to species adapted to being commensals of humans, of which some can evolve into opportunistic pathogens. A certain level of adaptation to the mammalian gut may have represented a selective advantage to yeast species that are naturally present in edible parts of plants (i.e. fruits), since this may have facilitated dispersion by the animals consuming the plants. Increased levels of adaptation to the mammalian gut environment may have resulted in species that persist in the gut and gradually adapt to a particular host (e.g. human). Once reached this point, particular features of some species may provide them with the ability to colonize an immuno-compromised host. Such features could be related to the ability to adhere to the host, persist in tissues other than the gut and to overcome the (debilitated) host immune system. Intriguingly, *C. nivariensis* seems not to be a inhabitant of the human gut, and may colonize human patients from an environmental source. Although further research is needed to clarify this, emergence of pathogenesis from environmental species would suggest that prior commensalism with humans is not a pre-requisite for developing infection capacity in *Candida spp*. Nevertheless, such species found in the environment may also be associated to other mammals. Clearly, additional data on ecological distribution of these *Nakaseomyces* species is needed to sort out these alternative hypotheses.

## Conclusions

Figure 3 shows a summary of the main findings described in this work. Comparative genomics analyses support the hypothesis that pathogenicity arose several times in the *Nakaseomyces*, and that this group represents a true genus, with common ancestral traits that may be favorable to adaptation to the human host.

## Methods

### Strains and DNA preparation

All strains are the type strains of the corresponding species [4,5,8,10,62,63]*,* obtained from the CBS collection. All media used were prepared as for *S. cerevisiae*: glucose and glycerol-based complete media, broth and solid. Cultures were performed at 30°C or 37°C, with agitation for broth cultures.

Anaerobic growth was tested by inoculating Sabouraud plates and using the Oxoid™ Anaerogen system [64]. Plates were examined after 120 h of incubation.

Petite mutants were obtained by exposing cells to Ethidium Bromide [65]. Petite mutants completely lacking mitochondrial (mt) DNA (ρ^0^ mutants) were used for the flow cytometry experiments so that mt DNA did not interfere with measures, since mt DNA content can be quite high in these species.

For the same reason, DNA for sequencing was prepared by standard zymolyase extraction followed by separation on a CsCl gradient with bis-benzimide [66]. The upper mt DNA band was discarded and the lower band used for sequencing. This procedure allowed the mt DNA sequence to be acquired nonetheless at an acceptable fold-coverage.

### Ploidy determination by flow cytometry

An aliquot of 4 mL from a fresh yeast culture at 1–2 10^6^ cells/mL is mixed with 9.2 mL of pure ethanol and incubated at 4C overnight. After centrifugation, cells are washed in 50 mM, pH7 Sodium Citrate and resuspended at 10^8^ cells/mL. An aliquot of 200 μL is treated with RNAse by adding 2 μL of a 100 mg/mL solution and incubating 2 hrs at 37°C. Half is then labeled by adding 400 μL of Propidium Iodide at 50 μg/mL in 50 mM Sodium Citrate, and incubating 20mn in the dark. The sample is then ready for the flow cytometer.

### Sequence and annotation of protein-coding genes

Sequencing was done by the Genoscope (Evry, France). Briefly, a whole genome shotgun (WGS) strategy associating different types of sequencing technologies was performed for each strain. An mean 24.8 genome equivalent (from 15.4 up to 41) was achieved using 454 GSFlx approach using a mixture of 8 kb mate pairs sequencing and single reads. Genome assembly was performed using 454 Newbler software. Subsequently, a mean 70 genome equivalent coverage for each strain was obtained using Illumina GAIIx technology 36 or 76 bp single reads, and these data were used to correct assembly errors [67]

Probably because of the complete sequence identity between segments of the three *MAT*-like cassettes, most of these loci were absent from the automated assembly generated from the Illumina and 454 reads. Only three loci were present in the assemblies: one *HMRa* from *C. bracarensis* and two *HMRa* from *C. nivariensis*. We therefore searched for sequence gaps in regions of synteny with the cassettes from *C. glabrata*. In all cases except one, there was indeed a gap in the region where the cassette was expected to be, by synteny conservation. The *HMR* cassette from *N. bacillisporus* is still missing from the assembly. Fragments were amplified by PCR and sequenced by Sanger sequencing.

Annotation of protein-coding genes was performed with an in-house procedure, which consists of two phases: syntactical annotation (prediction and location of protein coding genes), followed by functional annotation of each element based on comparison with known sequences. The first phase calls upon 6 gene prediction algorithms: CONRAD [68], AUGUSTUS [69], GETORF [70], SNAP [71], GENEMARK [72], GENEID [73], using the same training set of gene sequences, which contains genes with and without introns, for those which needed a training step; the intron-containing genes being defined by comparison to intron-bearing genes of *C. glabrata* and *S. cerevisiae*. All predictions as well as tBLASTn alignments to proteomes of reference species and Uniprot, and PSI-tBLASTn alignments to PSSM representative of Génolevures protein families are integrated using JIGSAW [74]. Then, all gene models from the 6 prediction algorithms plus JIGSAW are put together and filtered to eliminate gene models having unrealistic introns. The overlap conflicts between elements and validation of gene models are solved by taking into account predicted gene models, other chromosomal elements already validated, and similarity regions, strands and frames. The resulting gene models are then submitted to functional annotation, based on a decision tree inspired by previous semi-automated annotation projects held by the Génolevures Consortium (which used BLASTp alignments to proteomes of reference species and Uniprot).

Identification of centromeres was done by searching with fuzznuc from the Emboss suite [70], for sequences homologous to *S. cerevisia*e consensus centromere sequence, ie “[AG]TCA[TC][AG]TG[AC][TC]N(73,167)G[GT]N(7,15)TTCCGAA” [75], and by manually checking for conservation of synteny in case of multiple hits within a single scaffold. Telomeric repeats were searched for by using the repeat motif from *C. glabrata*, CAGCACCCAGACCCCA, as blastn queries against the genomic sequences. This also revealed the putative template inside the *TLC1* gene.

### ncRNA discovery and annotation

tRNA genes were identified by both cloverleaf structure detection [76] and tRNAscan-SE [77]. BLAST [78] and Infernal [79] searches were performed on each of the five *Nakaseomyces* genomes for other ncRNA genes; using annotated ncRNAs from the Genolevures database [80] as queries for BLAST, and the covariance models from RFam database [81] for the Infernal search. The hits of both searches were combined according to the respective ncRNA family and extended in order to detect contiguous hits corresponding to a same candidate. All hits from the same families were aligned and manually checked. Hits were accepted as candidates if: i) the sequence agrees with known structural features, guiding sequences (for snoRNAs) and conserved sequence motifs for homologous molecules and ii) known synteny was verified.

### Phylogenomics

A phylome, the complete collection of phylogenetic trees for each gene in a genome, was reconstructed for each one of the six *Nakaseomyces* species (five newly sequenced and the reference *C. glabrata* genome). The phylomes include 16 other species: *Saccharomyces cerevisiae, S. mikatae, S. (Naumovia) castellii, S. kluyveri, S. bayanus, Vanderwaltozyma polyspora, Lachancea thermotolerans, Ashbya gossypii, Candida dubliniensis, Kluyveromyces lactis, Candida albicans, Debaryomyces hansenii, Zygosaccharomyces rouxii, Yarrowia lipolytica, Pichia stipitis, Lachancea waltii.* Phylomes were reconstructed using the pipeline described in [82]. In brief, for all genes in each *Nakaseomyces* genome, a Smith-Waterman search [83] was used to retrieve homologs using an e-value cut-off of <10^-5^, and considering only sequences that aligned with a continuous region representing more than 50% of the query sequence.

Once the sets of homologous sequences were defined, phylogenetic trees were reconstructed as follows. Selected sequences were aligned using three different programs: MUSCLE v3.7 [84], MAFFT v6.712b [85], and DIALIGN-TX [86]. Alignments were performed in forward and reverse direction (i.e. using the Head or Tail approach [87]), and the six resulting alignments were combined using M-COFFEE [88]. The resulting combined alignment was subsequently trimmed with trimAl v1.3 [89] using a consistency score cutoff of 0.1667 and a gap score cutoff of 0.9.

The selection of the evolutionary model best fitting each protein alignment was performed as follows: A phylogenetic tree was reconstructed using a Neighbour Joining (NJ) approach as implemented in BioNJ [90]; The likelihood of this topology was computed, allowing branch-length optimisation, using seven different models (JTT, LG, WAG, Blosum62, MtREV, VT and Dayhoff), as implemented in PhyML v3.0 [91]. The two evolutionary models best fitting the data were determined by comparing the likelihood of the used models according to the AIC criterion [92]; Then, ML trees were derived using these two models. All trees and alignments have been deposited in PhylomeDB [82] and can be browsed on-line (http://www.phylomedb.org, phylome codes 78 to 83). Trees were scanned to i) define orthology and paralogy relationships using a phylogeny-based, species overlap approach [28]; ii) detect and date duplication events [29], including large expansions of gene families; and iii) transfer functional annotations from one-to-one orthologs in *S. cerevisiae*. Unless indicated otherwise, all operations with phylogenetic trees were performed using scripts implemented within the ETE package [50].

To reconstruct a species phylogeny, alignments of 603 proteins that had a single ortholog in all species considered in the phylome were concatenated into a single trimmed alignment of 288,995 positions. A Maximum Likelihood tree was reconstructed using phyML using the same parameters indicated for the trees in the phylome and the LG model. Branch support values were computed using the aLRT approach (see above) and based on an analysis of 100 bootstrap repetitions. In addition, a super-tree was reconstructed from the 4,965 gene phylogenies contained in the *N. delphensis* phylome, using a tree parsimony approach as implemented in DupTree [20]. Both approaches yielded identical topologies.

Average levels of sequence identity between orthologs were computed as follows: Each orthologous pair was aligned with MUSCLE v3.7 and the level of sequence identity was measured with trimAl V1.3 [89] as the number of identical residues over the length of the shortest protein.

### Substitution rate acceleration along specific lineages

In order to investigate how selective pressure varied along specific lineages in the phylogeny and whether positive selection was involved in the evolution and diversification of the *Nakaseomyces*, we used a subset of the original data. The subset analyzed included 7 species: *the 6 Nakaseomyces* and *S. cerevisiae* as outgroup. Following the same automated pipeline previously described to construct the *Nakaseomyces* phylome, we retrieved all the shared orthologous genes present in a single copy in all 7 genomes (i.e., one-to-one orthologs), we concatenated their respective alignments and estimated a phylogenetic tree using maximum likelihood. The resulting topology is consistent with that of the species tree in Figure 1. Using this phylogeny, we tested whether the rate of evolution of the one-to-one orthologs had accelerated along specific branches of the tree (i.e., affecting different species in the group), which would be consistent with either the relaxation of selective constraints or with the action of positive selection. We used the program codeml in the paml 4 package [93] to estimate the *d*_*N*_/*d*_*S*_ rate ratio variation in each individual one-to-one ortholog, along particular branches in the tree. Subsequently, we compared pairs of nested models by means of a likelihood ratio test (LRT) where the degrees of freedom correspond to the difference in the number of parameters estimated in each model, and the distribution of the *d*_*N*_/*d*_*S*_ rate is assumed to follow a chi square distribution. Values of d_N_/d_S_ larger than 5 were filtered out.

We hypothesized an acceleration in gene evolution rate, and possible cases of positive selection, preceding the diversification of the *‘glabrata* group’, that were perhaps involved in the capability of these species to become pathogenic. In LRT A (Additional file 12) we compare: *i*) A model that assumes an overall *d*_*N*_/*d*_*S*_ rate ratio for all branches in the tree (omega 1) and a different rate for the branch that is ancestral to the *‘glabrata* group’ (omega 2), where we hypothesize a rate acceleration; and *ii*) an alternative model that assumes one *d*_*N*_/*d*_*S*_ ratio for the basal branches including *S. cerevisiae, C. castellii* and *N. bacillisporus* (omega 1), a different *d*_*N*_/*d*_*S*_ rate in the branch ancestral to the *‘glabrata* group’ (omega 2), and another *d*_*N*_/*d*_*S*_ rate for the genus itself (omega 3). In this test, we were interested in verifying whether there was a significant increase in the *d*_*N*_/*d*_*S*_ rate in the branch ancestral to the *‘glabrata* group’ relative to the overall rate and whether this increase also occurred in the different species within the group.

To investigate whether pathogenic and non-pathogenic species were subjected to different selective pressure, we built two more LRTs to analyse another subset of species focusing on the *‘glabrata* group’, we therefore excluded *S. cerevisiae*, and *C. castellii*, and used *N. bacillisporus* as the outgroup (Additional file 12). In LRT B we compared *i*) a model with two rates, one for the *‘glabrata* group’, and one for the outgroup (*N. bacillisporus*); with *ii*) a model with four rates, one for the pathogenic species, one for the single non-pathogenic species in the subset (*N. delphensis*)*,* one for the branch ancestral to the *‘glabrata* group’, and one for the outgroup. In LRT C we compared *i*) a model with two rates, one for the *‘glabrata* group’, and one for the outgroup (*N. bacillisporus*); with *ii*) a model with four rates, one for the the *‘glabrata* group’, one for the branch ancestral to the *‘glabrata* group’, one for the *C. glabrata* species itself and one for the outgroup.

### Detection of adhesin genes

Putative adhesins were detected in the newly sequenced *Nakaseomyces* using a similarity search based on the known *EPA* genes in *C. glabrata* and the *FLO* genes in *S. cerevisiae*. Hits were filtered using the same thresholds applied during phylome reconstruction. Additionally, all proteomes were scanned for the presence of the Pfam domain PF10528, which is related to adhesins in fungi. The search was performed using HMMER v3, and proteins containing this domain with an e-value below 1e-05 were added to the selection of adhesins. Proteins were then scanned for undetermined regions as their sub-telomeric location can cause problems in the assembly. Proteins containing more than 33% of undetermined regions were excluded from further analysis (this affected 8 proteins, of which 1 in *C. castellii*, 2 in *N. delphensis*, 4 in *C. bracarensis*, and 1 in *C. nivariensis*). Putative adhesins were then clustered using the TribeMCL [94] algorithm as implemented in scps (inflation = 1.5) [95]. Clusters were then used to infer phylogenetic trees. First the repetitive regions of each sequence were masked using SEG [96]. Alignments were then reconstructed using MUSCLE v3.7 [84] followed by a maximum likelihood tree reconstruction as implemented in PhyML. The LG model was used along with four rate categories and invariant positions infered from the data.

## Competing interests

The authors declare that they have no competing interests.

## Authors’ contributions

TG and CF analysed genome data and wrote the paper. AC, EP, JP, PW produced genome data. TM, PD produced tools for automatic genome annotation and analysis. OL produced tools for genome analysis and analysed genome data. SA, SB, LJ, AP, JR produced and analysed *MAT* cassettes data. RA, SA, LJ, CF produced PFGE and ploidy data. MBF, MMH, GA, RA, CB, SC, JAC, HD, AEA, JG, LM, CM, CN, EW, BD, CH analysed genome data. All authors read and approved the final manuscript.

## Supplementary Material

Additional file 1 Assembly data. Click here for file

Additional file 2 **Flow cytometry of the *Nakaseomyces.*** Species names are indicated above each panel. 1C, 2C, 4C indicate peaks corresponding to the DNA content of, respectively, one, two and four haploid genomes. Click here for file

Additional file 3 **Supplementary text containing additional results and references. Table S1.** tRNA genes in the *Nakaseomyces* genomes. **Table S2.** GC inserts in the mitochondrial genomes of *C. bracarensis* and *C. nivariensis*. Click here for file

Additional file 4 **Pair-wise species identity between orthologous protein-coding genes.** Each histogram represents the numbers of orthologous gene pairs according to their percentage of identity. Click here for file

Additional file 5 **Number of synteny blocks according to the mean length of synteny blocks.** All pairwise comparisons of the genomes of the *Nakaseomyces* and *S. cerevisiae* (out-group) are shown. Genomes were divided into three groups according to their location in the phylogenetic tree (Figure 1): the *‘glabrata* group’ (red); *C. castellii* and *N. bacillisporus* (green); *S. cerevisiae* (blue). Each dot corresponds to one pairwise comparison and is colored according to the groups of the two compared genomes. Click here for file

Additional file 6 **Alignment of putative Ho proteins.** “LAGLIDADG” motifs and Nuclear Localization Signals are boxed. Click here for file

Additional file 7 **Histograms of *Nakaseomyces* genes according to the length of encoded proteins.** Click here for file

Additional file 8 **Genes from *S. cerevisiae* that are absent from *C. glabrata* and their absence/presence in the *Nakaseomyces.*** Click here for file

Additional file 9 **Glycolytic enzymes in the *Nakaseomyces.*** Click here for file

Additional file 10 **The *DAL* cluster.** The cluster from *S. cerevisiae* is shown at top. The cluster containing the additional *DAL5* gene in *C. castellii* and *N. bacillisporus* is shown below, using the gene nomenclature from *S. cerevisiae*. In these two genomes, the cluster differs only by the synteny on the left. Genes are represented by arrows, genes in black are *DAL* genes. Click here for file

Additional file 11 **Duplicated ribosomal protein genes in the *Nakaseomyces.*** Click here for file

Additional file 12 Scenarios for positive selection tests. Click here for file
