# Supplementary figures and images for: Comparative genomics of emerging pathogens in the Candida glabrata clade
Source: BMC Genomics. 2013 Sep 14;14:623. doi: 10.1186/1471-2164-14-623 (PMC3847288; doi:10.1186/1471-2164-14-623)

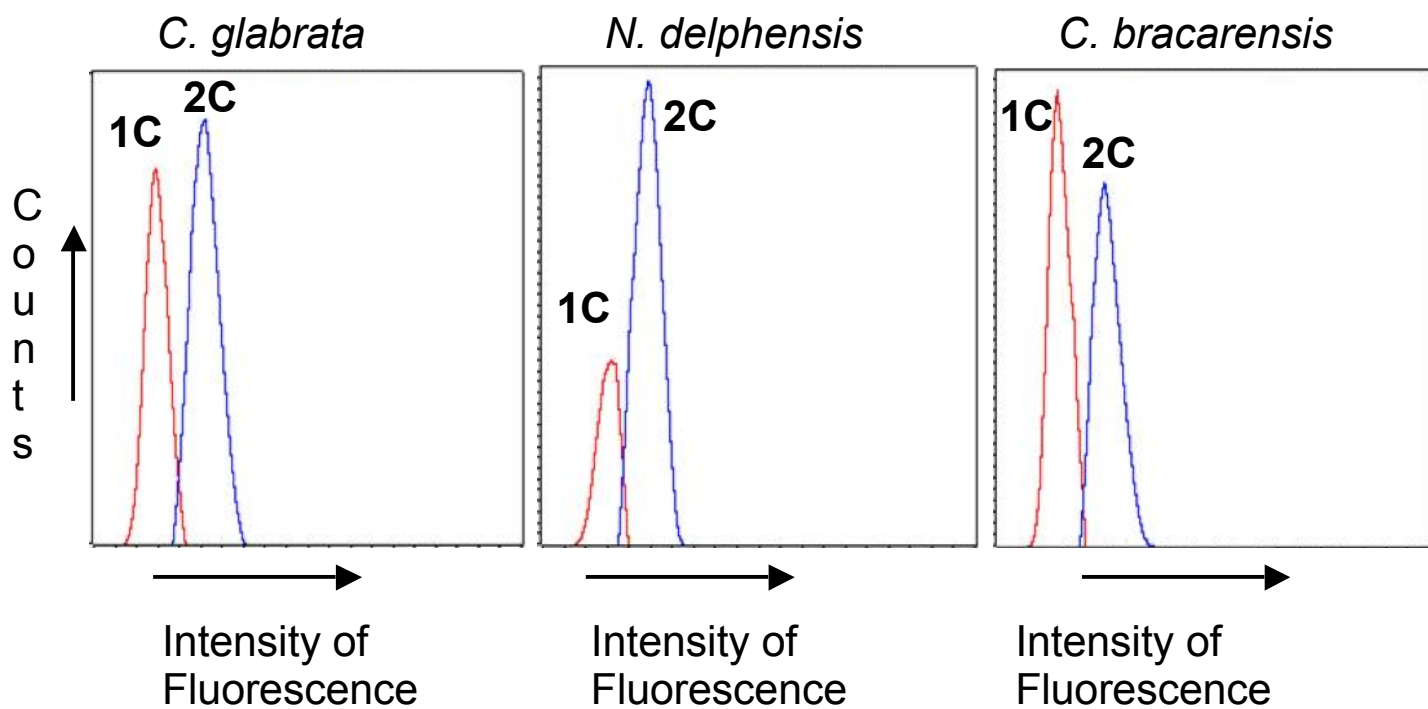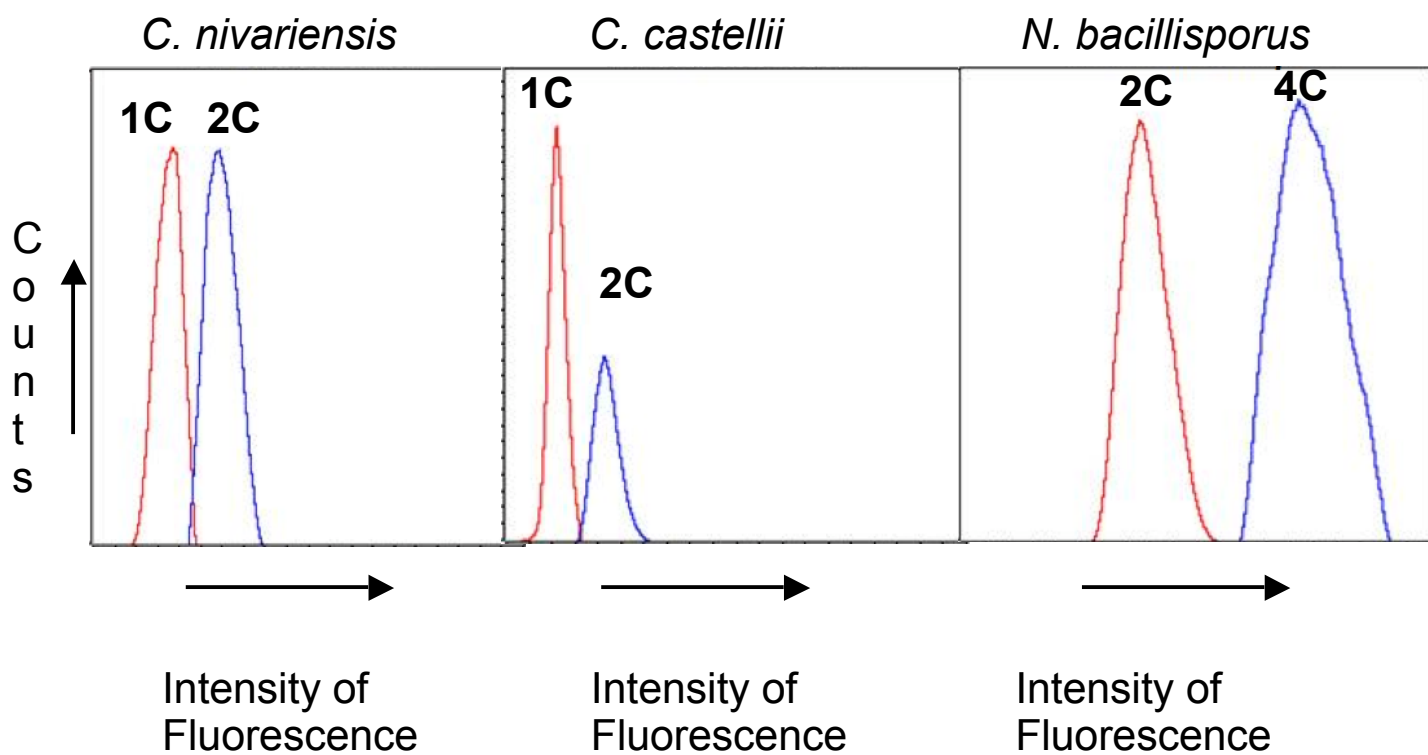

Supplement: Additional file 2 — Flow cytometry of the Nakaseomyces. Species names are indicated above each panel. 1C, 2C, 4C indicate peaks corresponding to the DNA content of, respectively, one, two and four haploid genomes. [file 1471-2164-14-623-S2.pdf]

*S. cerevisiae*

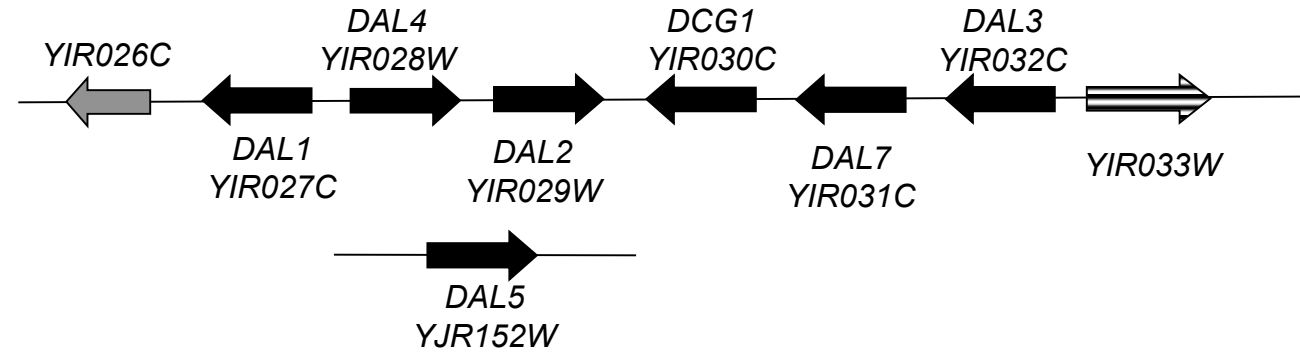

*N. bacillisporus*  
Scaffold 29  
931K-945K

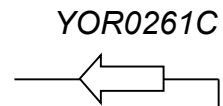

*C. castellii*  
Scaffold 5  
1666K-1650K  
(reverse)

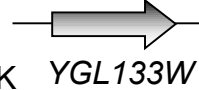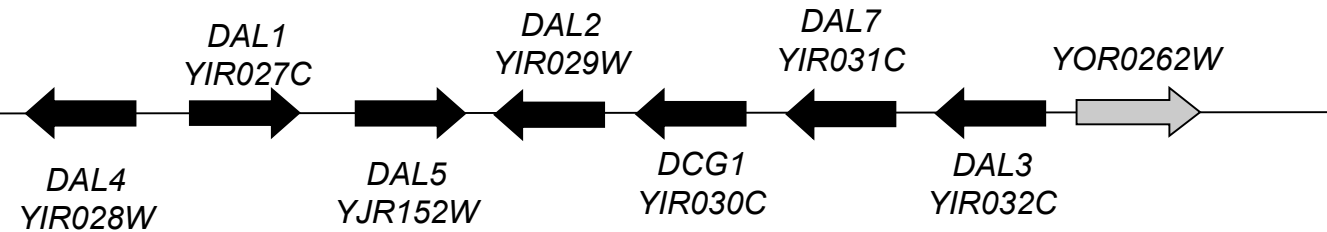

Drawing not to scale

Supplement: Additional file 10 — The DAL cluster. The cluster from S. cerevisiae is shown at top. The cluster containing the additional DAL5 gene in C. castellii and N. bacillisporus is shown below, using the gene nomenclature from S. cerevisiae. In these two genomes, the cluster differs only by the synteny on the left. Genes are represented by arrows, genes in black are DAL genes. [file 1471-2164-14-623-S10.pdf]
