# Supplementary material for: Comparative genomics of emerging pathogens in the Candida glabrata clade
Source: BMC Genomics. 2013 Sep 14;14:623. doi: 10.1186/1471-2164-14-623 (PMC3847288; doi:10.1186/1471-2164-14-623)
Supplement: Additional file 3 — Supplementary text containing additional results and references. Table S1. tRNA genes in the Nakaseomyces genomes. Table S2. GC inserts in the mitochondrial genomes of C. bracarensis and C. nivariensis. [file 1471-2164-14-623-S3.doc]

**ADDITIONAL FILE 3:**

**SUPPLEMENTARY RESULTS**

**Distribution of gene sizes and protein divergence**

Distribution of gene sizes is similar across the newly-sequenced Nakaseomyces, with the exception of the class of shorter proteins, which is more numerous in the newly sequenced genomes compared to *C. glabrata* and *S. cerevisiae* (Additional file 7). This is probably due to the more extensive curation in these two species, which has likely discarded false positives from the automated annotations. We took this into account for the comparison of total numbers of coding genes (see main text). In addition, we compared the distribution of sequence identities at the protein level in all possible species pairs. As shown in Additional file 4, all distribution were unimodal and centered around the mean protein identity.

**Nc RNA genes**

Types and numbers of tRNA genes are shown in Additional file 3: Table S1. Total number of tRNA genes ranges from 138 to 236. *C. nivariensis*, *C. bracarensis* and *N. delphensis* share the same 42- tRNA gene set as *C. glabrata* and *S. cerevisiae*; but both *C. castellii* and *N. bacillisporus* have an additional tRNA-Ala (CGC) gene. This gene is present in more distant species such as *Debaryomyces* *hansenii*. Presence of an intron in a given tRNA is conserved across species and identical to *C. glabrata*, except for the particular tRNA-Leu (GAG), involved in the frameshift readthrough phenomenon linked to translation of the *EST3* and *ABP140* genes. This gene has an intron in *C. castellii* and *N. bacillisporus* only. Note that this rare intron acquisition event supports the topology depicted in Figure 1.

All of the small nuclear RNAs (U1, U2, U4, U5 and U6), both RNA components of the RNase P, and the signal recognition particle were found in all genomes. 117 H/ACA and 201 C/D snoRNAs were also found, corresponding to 90% of all known yeast snoRNAs. Telomeric repeats identical to those in *C. glabrata* were found at ends of some scaffolds, only in *N. delphensis*, *C. nivariensis* and *C. bracarensis*, and indeed the putative telomerase RNA-component, containing the template for telomere elongation has also been identified in these three species, with a template identical to *C. glabrata*'s, and in the same location. Notably, the snR161 H/ACA snoRNA gene position is conserved about 1kb from *TLC1*, in the opposite strand, as it is in *S. cerevisiae*, in all species where we identified *TLC1*. It is likely that *C. castellii* and *N. bacillisporus* have divergent telomeric repeats and thus use a different template.

**Mitochondrial genomes**

The mitochondrial (mt) genomes of *C. glabrata*, *N. delphensis*, *C. castellii* and *N. bacillisporus* were already sequenced (main text and [1]). The mt genomes of *C. bracarensis* and *C. nivariensis*, respectively 44.6 and 28.5 kb-long, exhibit the same set of protein-coding genes as all species from the post WGD *Saccharomyces*, ie *VAR1*, *CYB*, *COX1*, *COX2*, *COX3*, *ATP6*, *ATP8* and *ATP9*.

All, except *C. glabrata*, contain small, palindromic GC clusters, thought to be related to mobile elements (Additional file 3: Table S2). Both mt genomes of *C. bracarensis* and *C. nivariensis* posess 40 to 50 such GC inserts. Notably, *C. glabrata* remains the species with the smallest mt genome (ca. 20 kb), and the only one without GC inserts, while the largest (100kb) is that of *N. bacillisporus*.

.

**Gene loss and gain**

As mentioned in the main text, comparative analysis of *C. glabrata*'s genome had revealed several gene losses and amplifications that were specific to this species as compared to *S. cerevisiae* (figures 3 and 4). We looked specifically at these, in order to determine if these events were ancestral to the *Nakaseomyces* or specific to the ‘*glabrata* group’ or to *C. glabrata* itself. Additional file 8 details some of the gene losses in the *Nakaseomyces* relative to *S. cerevisiae*.

Two cases of gene loss were found because they represent specific losses within a pair of genes: the *TOP1* gene encoding topoisomerase I (*YOL006C*), unique in *S. cerevisiae*, is duplicated in *C. glabrata* [2] and the *'glabrata* group' but not in *C. castellii* or *N. bacillisporus*; and the *PAM18* (*YLR008C*) / *MDJ2* (*YNL328C*) pair, involved in import of proteins into the mitochondrion and which dates back to the *Saccharomycotina*/ *Pezizomycotina* split [3], has only a single homolog in *C. castellii* and *N. bacillisporus*.

*C. glabrata* lacks the dihydroorotate dehydrogenase gene *URA1* present in *S. cerevisiae* and shown to have been transferred from lactic bacteria [4]. Acquisition of *URA1*, was followed by loss of the original dihydroorotate dehydrogenase gene (*URA9*) in many species including *S. cerevisiae*, while other species lost the newly acquired gene without losing *URA9* (e.g. *C. glabrata*), or kept the two *URA* genes (e.g. *Kluyveromyces* *lactis*). We found that most of the newly sequenced *Nakaseomyces* resemble *C. glabrata* in that they only contain *URA9*, with the exception of *N. bacillisporus* that has both genes, suggesting two independent recent losses of *URA1* within the *Nakaseomyces*.

Cases of gene gains include another gene involved in DNA metabolism, *SGS1*, encoding a helicase of the *RecQ* family [5]. This gene is single-copy in *S. cerevisiae*, but duplicated in *C. glabrata* [2], as in all *Nakaseomyces*.

**Regulation of the translational machinery**

Crf1 is a regulator of ribosomal proteins (RP) in *S. cerevisiae*, repressing their expression under stress conditions. The gene encoding Crf1 is absent in *C. glabrata*, a fact associated with the scarcity or RP proteins in this species [6]. The other *Nakaseomyces* species have also lost this gene, with the possible exception of a degenerate homolog found in *C. castellii*. The number of conserved RP genes differs in the Nakaseomyces species (Additional file 11). *S. cerevisiae* contains 17 RP proteins that have maintained their ohnolog after the WGD, whereas *C. glabrata* was predicted to have maintained five pairs of conserved RP [6]. We identified two new ohnologs, increasing the number of duplicated RP to seven in *C. glabrata*. These RP ohnologs are conserved in the *'glabrata* group' while *C. castellii* and *N. bacillisporus* contain different sets of duplicated RP genes, though always pairs that were also found in *S. cerevisiae*. *N. bacillisporus* represents an interesting case, since, as the other *Nakaseomyces*, it has lost the Crf1 protein and yet it contains 13 ohnologous RP gene pairs, a high number considering the lack of the stress-induced repressor. An additional feature that singles *N. bacillisporus* out is the lack of duplication of *SFP1*. This gene, present in single-copy in *S. cerevisiae*, has also been associated to RP regulation. It was found duplicated in *C. glabrata*, the distant *Naumovia* (*Saccharomyces*) *castellii*, and the *Nakaseomyces* with the exception of *N. bacillisporus*.

**Central carbon metabolism**

The *Nakaseomyces* clade is supposed to share most specific metabolic traits with *S. cerevisiae*; such as, i) a fermentative metabolism (i.e. the Crabtree effect: repression of respiratory enzymes by high fermentation rates), ii) the possibility to grow in anaerobiosis, and iii) the ability to generate "petite" mutants. We nonetheless observe many variations in gene repertoires, as mentioned in main text. Genes encoding the respiratory chain and enzymes of the Krebs cycle are present in similar numbers to *S. cerevisiae*. Similar to other *Saccharomycetes* [7], all *Nakaseomyces* lack the NADH:Ubiquinone oxidoreductase complex except for the Ndufab1 subunit. Regarding carbohydrate utilization, *C. glabrata* and all *Nakaseomyces* are gal-, i.e. unable to assimilate galactose, and indeed, we confirm the absence of the *GAL* genes in all genomes. The situation is less clear for the utilization of lactic acid, since *C. glabrata*'s phenotype is described as strain-dependent. Nonetheless, the type strain is lactate+ and it has been hypothesized that hypoxic lactate utilization may be important for human gut residents, as oxygen and glucose may be in limited supply, whereas lactate is produced by intestinal bacteria [8]. Lactate utilization in such conditions has recently been shown to depend on the L-lactate dehydrogenase activity encoded by the *CYB2* gene in *C. glabrata* [8]. This remains to be proven for the other *Nakaseomyces*. In *S. cerevisiae*, the D-lactate dehydrogenase activity is encoded by three genes (*DLD1*-*3*), but *C. glabrata*, as well as all other *Nakaseomyces*, possess only two such gene copies. Finally, none of the *Nakaseomyces* contain a homolog of *JEN1* (encoding a lactate and monocarboxylic acid transporter), but, intriguingly, recent work in *S. cerevisiae* mutants lacking the *JEN1* gene has shown that the acetate transporter gene *ADY2* can evolve to overtake *JEN1* function [9].

**SUPPLEMENTARY REFERENCES**

1. Koszul R, Malpertuy A, Frangeul L, Bouchier C, Wincker P, Thierry A, Duthoy S, Ferris S, Hennequin C, Dujon B: **The complete mitochondrial genome sequence of the pathogenic yeast *Candida* (*Torulopsis*) *glabrata***. *FEBS Letters* 2003, **534**:39-48.

2. Richard GF, Kerrest A, Lafontaine I, Dujon B: **Comparative genomics of hemiascomycete yeasts: genes involved in DNA replication, repair, and recombination.** *Mol Biol Evol* 2005, **22**:1011-1023.

3. Hayashi M, Schilke B, Marszalek J, Williams B, Craig EA: **Ancient gene duplication provided a key molecular step for anaerobic growth of Baker's yeast.** *Mol Biol Evol* 2011, **28**:2005-2017.

4. Gojkovic Z, Knecht W, Zameitat E, Warneboldt J, Coutelis JB, Pynyaha Y, Neuveglise C, Moller K, Loffler M, Piskur J: **Horizontal gene transfer promoted evolution of the ability to propagate under anaerobic conditions in yeasts.** Mol Genet Genomics 2004, **271**:387-393.

5. Gangloff S, McDonald JP, Bendixen C, Arthur L, Rothstein R: **The yeast type I topoisomerase Top3 interacts with Sgs1, a DNA helicase homolog: a potential eukaryotic reverse gyrase.** *Mol Cell Biol* 1994, **14**:8391-8398.

6. Wapinski I, Pfiffner J, French C, Socha A, Thompson DA, Regev A: **Gene duplication and the evolution of ribosomal protein gene regulation in yeast.** *Proc Natl Acad Sci USA* 2010, **107**:5505-5510.

7. Marcet-Houben M, Marceddu G, Gabaldon T: **Phylogenomics of the oxidative phosphorylation in fungi reveals extensive gene duplication followed by functional divergence.** *BMC Evol Biol* 2009, **9**:295.

8. Ueno K, Matsumoto Y, Uno J, Sasamoto K, Sekimizu K, Kinjo Y, Chibana H: **Intestinal resident yeast *C*. *glabrata* requires Cyb2p-mediated lactate assimilation to adapt in mouse intestine.** *PLoS One* 2011, **6**:e24759.

9. de Kok S, Nijkamp JF, Oud B, Roque FC, de Ridder D, Daran JM, Pronk JT, van Maris AJ: **Laboratory evolution of new lactate transporter genes in a jen1Delta mutant of *S. cerevisiae* and their identification as *ADY2* alleles by whole-genome resequencing and transcriptome analysis.** *FEMS Yeast Res* 2012, **12**: 359–374.

**Additional file 3:Table S1. tRNA genes in the *Nakaseomyces* genomes**

"+": tRNA gene with intron

"§": tRNA-Ala (CGC) present only in *C. castellii* and *N. bacillisporus*

"*": special tRNA-Leu (GAG) responsible for +1 frameshift of *ABP140* (*YOR239W*) and *EST3* (*YIL009C-A*) homologs

AA C AC C. C. C. N. N.

bracarensis castellii nivariensis bacillisporus delphensis

------ --- --- -------- -------- -------- -------- --------

F Phe TTT --- - - - - -

F Phe TTC (GAA) 8+ 5+ 8+ 4+ 6+

L Leu TTA (TAA) 3 2 3 6 3

L Leu TTG (CAA) 9 5 10 4 7

L Leu CTT (AAG) - - - - -

L Leu CTC (GAG) 1 1+ 1 1+ 1 *

L Leu CTA (TAG) 4+ 2+ 4+ 1+ 4+

L Leu CTG (CAG) - - - - -

I Ile ATT (AAT) 11 6 11 7 10

I Ile ATC (GAT) - - - - -

I Ile ATA (TAT) 2+ 1+ 2+ 2+ 2+

M Met ATG (CAT) 4 3 4 3 4

m iMet ATG (CAT) 5 3 4 3 4

V Val GTT (AAC) 12 6 13 10 10

V Val GTC --- - - - - -

V Val GTA (TAC) 2 1 2 2 2

V Val GTG (CAC) 1 2 1 3 1

S Ser TCT (AGA) 9 5 10 6 9

S Ser TCC --- - - - - -

S Ser TCA (TGA) 2 1 2 2 2

S Ser TCG (CGA) 1+ 1+ 1+ 1+ 1+

P Pro CCT (AGG) 1 2 1 1 1

P Pro CCC --- - - - - -

P Pro CCA (TGG) 8+ 5+ 9+ 7+ 7+

P Pro CCG (CGG) - - - - -

T Thr ACT (AGT) 9 5 10 5 8

T Thr ACC --- - - - - -

T Thr ACA (TGT) 3 2 3 2 3

T Thr ACG (CGT) 1 1 1 1 1

A Ala GCT (AGC) 11 5 12 7 4

A Ala GCC --- - - - - -

A Ala GCA (TGC) 5 2 5 4 5

A Ala GCG (CGC) - 1 - - 1 §

AA C AC C. C. C. N. N.

bracarensis castellii nivariensis bacillisporus delphensis

------ --- --- -------- -------- -------- -------- --------

Y Tyr TAT --- - - - - -

Y Tyr TAC (GTA) 5+ 4+ 6+ 5+ 6+

* Och TAA --- - - - - -

* Amb TAG --- - - - - -

H His CAT --- - - - - -

H His CAC (GTG) 6 4 6 4 6

Q Gln CAA (TTG) 7 4 7 7 6

Q Gln CAG (CTG) 2 2 2 1 2

N Asn AAT --- - - - - -

N Asn AAC (GTT) 8 5 9 7 5

K Lys AAA (TTT) 3+ 3+ 3+ 6+ 3+

K Lys AAG (CTT) 13 6 14 9 7

D Asp GAT --- - - - - -

D Asp GAC (GTC) 12 7 12 10 11

E Glu GAA (TTC) 11 4 11 9 10

E Glu GAG (CTC) 4 4 4 2 4

C Cys TGT --- - - - - -

C Cys TGC (GCA) 4 3 4 3 4

* Opa TGA --- - - - - -

W Trp TGG (CCA) 5+ 3+ 5+ 4+ 5+

R Arg CGT (ACG) 4 3 4 3 4

R Arg CGC --- - - - - -

R Arg CGA (TCG) - - - - -

R Arg CGG (CCG) 1 1 1 1 1

S Ser AGT --- - - - - -

S Ser AGC (GCT) 3+ 2+ 3+ 3+ 3+

R Arg AGA (TCT) 9 4 10 7 9

R Arg AGG (CCT) 1 1 1 1 1

G Gly GGT --- - - - - -

G Gly GGC (GCC) 13 8 14 9 12

G Gly GGA (TCC) 2 3 2 2 2

G Gly GGG (CCC) 1 1 1 1 1

__________________________________________________________________________

Number of tDNA 226 138 236 175 198

Nb of anticodons 42 43 42 42 43

__________________________________________________________________________

**Additional file 3: Table S2. GC inserts in the new mt genomes.**

GC inserts were searched by examining each occurrence of the triplet "CCC" in the mt genomes

sequenced in this project: C. bracarensis' and C. nivariensis'. Sequences of palindromes found are listed.

| **GC inserts detected in mt genome of C. nivariensis** | **GC inserts detected in mt genome of C. bracarensis** |
| --- | --- |
| ccccataataccttatataataaggtattatgggg | cccggcataacgaggtaacacacatttctcttacctgtatatgccggg |
| ccccataataccttcttataaagaaggtattatgggg | cccgcatatatgaggtaaacacataagtgtttaccttatt |
| ccccataatcctatattataggattatggg | ccccataataataccacttaataagtggtattattatgggg |
| ccccagaatccctataatatagggattctggg | ccccatataatacctatataatatttggtattatatggggg |
| ccccccccaataatccaatagatattggattattgggg | ccccgaggctatgcctcgggg |
| cccagaaccctctaaaagagggttctggg | ccccgaggcatagcatagcctcgggg |
| ccccagaatctctatataatagagattctggg | ccccgaggtataacctcggggg |
| ccccctccacatttactttgtaaatgaggaggggg | ccccccccccccgagatatcggggggggggggg |
| ccccctcctcagatatatctgaggaggggg | ccccgataacattgggttatcgggg |
| cccccccccatccagatatacaaaatatatctggatgggg | ccccgataacccgaggttatcgggg |
| cccccccccccataatcctctatatagaggattatggggggggggg | ccccgaggtataacctcgggg |
| ccccacccatatactgatgtatatgggtgggg | cccgaggtataacctcggg |
| ccccacccatatacaaatgtatatgggtgggg | cccccaccattacataatattatcatatcatgtaatggtggg |
| ccccacccatatactaatgtatatgggtgggg | cccggatataccaggtaattcttatcctggtatagccggg |
| cccacccatcatatattctatatgatgggtggg | ccccaccataaccaggtaacacctagatggtgttacctgtatatggtgggg |
| cccacccatcatataaaatatgatgggtggg | cccgccatatacatgtatatgtatacctgtatatggcggg |
| cccacccatatacaaatgtatatgggtggg | cccgcctaatctaattgaggtaacatttatatgttacctcatatagatataggcggg |
| ccccactcatgtactaatgtacatgagtgggg | cccgcatatacaggtatacatgtatatgtatacctggttatgcggg |
| ccccactcatatactaatgtatatgagtgggg | ccccaccataaccaggtaacaccaatatggtgttacctgtatatggtgggg |
| ccccactcagatacattagtatctgagtgggg | ccccacccataaccaggtaatacctatatggtattacctgtatatggtggggg |
| ccccactcatatacaagtgtatatgagtgggg | ccccatcataaccaggtaataccctacgggtattacctgtatatgatgggg |
| cccatcatcatcattaatgatgatgatggg | cccccatataaccaggtaaacatatacatgtttacctgtatatatggggggggg |
| cccacccatatactattgtatatgggtggg | ccccacataacaaggtaaacatttacatgtttacctatatatgtgggg |
| cccacccaattatactaatgtataatgggtggg | ccccaccatatacaggtatacatgtatatgtatacctggttatggtgggg |
| cccacccatctacatttgtagatgggtggg | ccccactatatacaggtatacatgtatatgtatacctggttatagtgggg |
| cccagccatatacaaatgtatatggctggg | cccatataatatcatatgtatatatgatattatagggg |
| cccacccttatacattagtataagggtggg | cccagtatatattcttatatatcatatagaatatatactggg |
| cccaccattatacaaatgtataatggtggg | cccccacataatatcatatgatatatgatattatgtgggg |
| ccccacccatataccttagtatatgggtgggg | cccccccccctaccccatataatatcctatatgtataaggatattatatgggg |
| ccacccatatactaatgtatatgggtgg | cccccccatataatatcattatagaatatatgatattatatgggg |
| ccccatcagatatatattaatatatctgatggg | ccccccaggtaatatcattatattatatggtattacctgggggg |
| cccacccatcatatagaatatatgatgggtggg | ccccgaggcatagcctcgggg |
| cccccattagatattaatatattaatatctaatggg | cccccccaaggtataccttggggggg |
| ccccatcagatatatattaatatatctgatgggg | ccccatgtaatatcatatatatataatgatattacatgggggg |
| cccacccatcatcatattatatggtgatgggtgggg | ccccaccatatactcatataatatatgagtatattgtgggg |
| ccccacctcatatatatacctatatatgatgtgggg | ccccaccatatattcatatatatctaatatatgaatatatagtgggg |
| cccctcctcatatactttgtatatgtggaggggg | ccccacaatatatccatatataaatatggatatattgtgggg |
| cccacccatcatcatattatgatgatgggtggg | cccacacttaatagcaggtaaacatgtatatgtttacctgtatataagtgtggg |
| cccctcctcatatacaaagtatatgtggagggg | cccgcgtaatatcatatatatatatgatattacacggg |
| ccccacctcatatatatgtatatatatgaggtgggg | cccaatagatacaaaatatctattggg |
| ccctgatctactatattagtagatcaggg | ccccacaatatatacatatattatatgtatatattgtgggg |
|  | cccaccataatgacatatcataaatgtcattatagtgggggg |
|  | ccccacaataattacatatattatatgtaattattgtgggg |
|  | ccccataataattacatatcacatatgtaattattatggggggggg |
|  | ccccataataataacatatagtatatgttattattatgggg |
|  | ccccgactagttcaggtaacatgtaaatgttacctgacataggcgggg |
|  | cccccgactagttcaggtaacatatacatgttacctgacataggcgggg |
