# Supplementary material for: Comparative genomics of emerging pathogens in the Candida glabrata clade
Source: BMC Genomics. 2013 Sep 14;14:623. doi: 10.1186/1471-2164-14-623 (PMC3847288; doi:10.1186/1471-2164-14-623)
Supplement: Additional file 4 — Pair-wise species identity between orthologous protein-coding genes. Each histogram represents the numbers of orthologous gene pairs according to their percentage of identity. [file 1471-2164-14-623-S4.pdf]

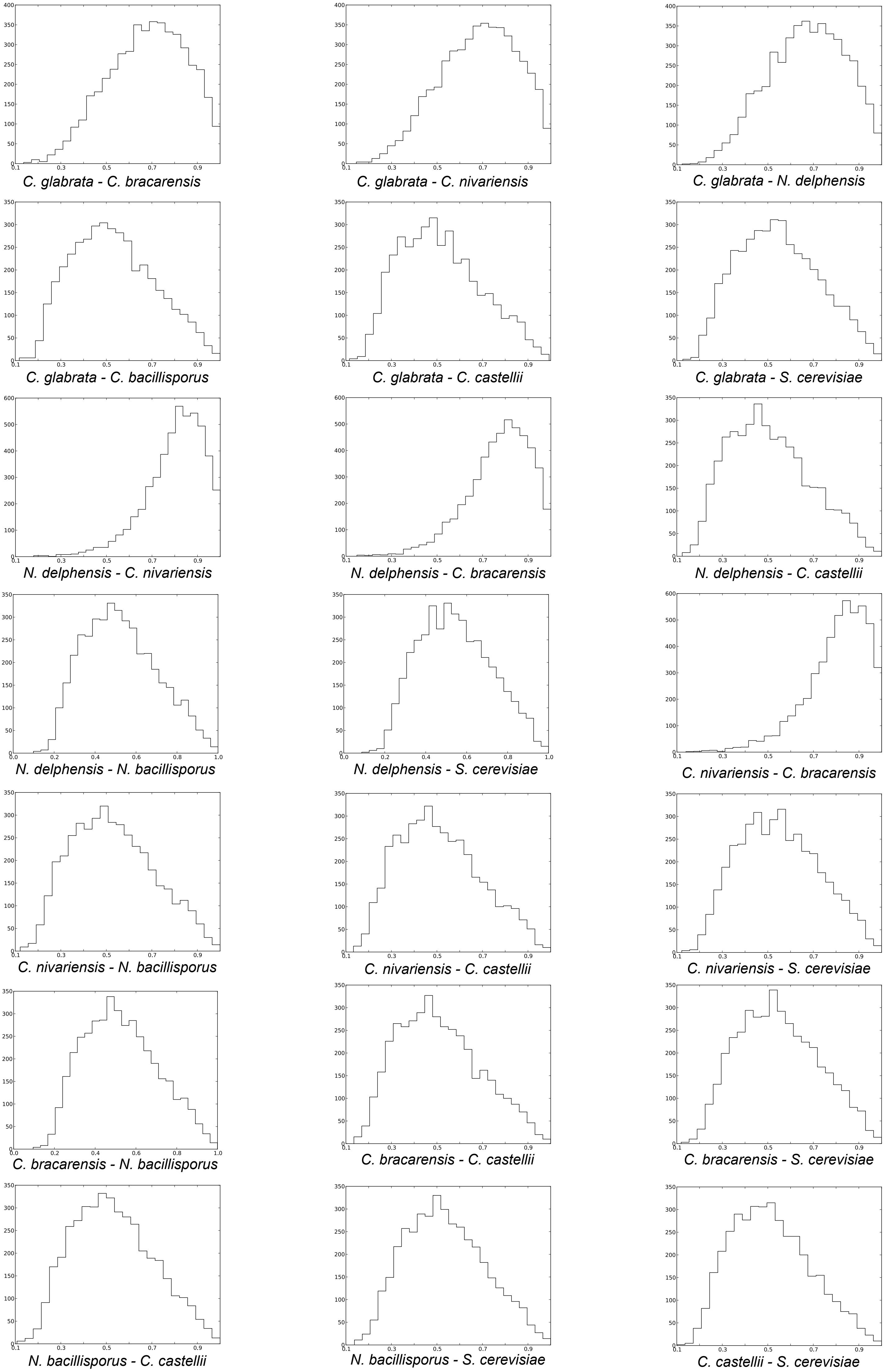
C. glabrata - C. bracarensis
C. glabrata - C. nivariensis
C. glabrata - N. delphensis
C. glabrata - C. bacillisporus
C. glabrata - C. castellii
C. glabrata - S. cerevisiae
N. delphensis - C. nivariensis
N. delphensis - C. bracarensis
N. delphensis - C. castellii
N. delphensis - N. bacillisporus
N. delphensis - S. cerevisiae
C. nivariensis - C. bracarensis
C. nivariensis - N. bacillisporus
C. nivariensis - C. castellii
C. nivariensis - S. cerevisiae
C. bracarensis - N. bacillisporus
C. bracarensis - C. castellii
C. bracarensis - S. cerevisiae
N. bacillisporus - C. castellii
N. bacillisporus - S. cerevisiae
C. castellii - S. cerevisiae
