# Supplementary material for: Comparative genomics of emerging pathogens in the Candida glabrata clade
Source: BMC Genomics. 2013 Sep 14;14:623. doi: 10.1186/1471-2164-14-623 (PMC3847288; doi:10.1186/1471-2164-14-623)
Supplement: Additional file 5 — Number of synteny blocks according to the mean length of synteny blocks. All pairwise comparisons of the genomes of the Nakaseomyces and S. cerevisiae (out-group) are shown. Genomes were divided into three groups according to their location in the phylogenetic tree (Figure 1): the ‘glabrata group’ (red); C. castellii and N. bacillisporus (green); S. cerevisiae (blue). Each dot corresponds to one pairwise comparison and is colored according to the groups of the two compared genomes. [file 1471-2164-14-623-S5.pdf]

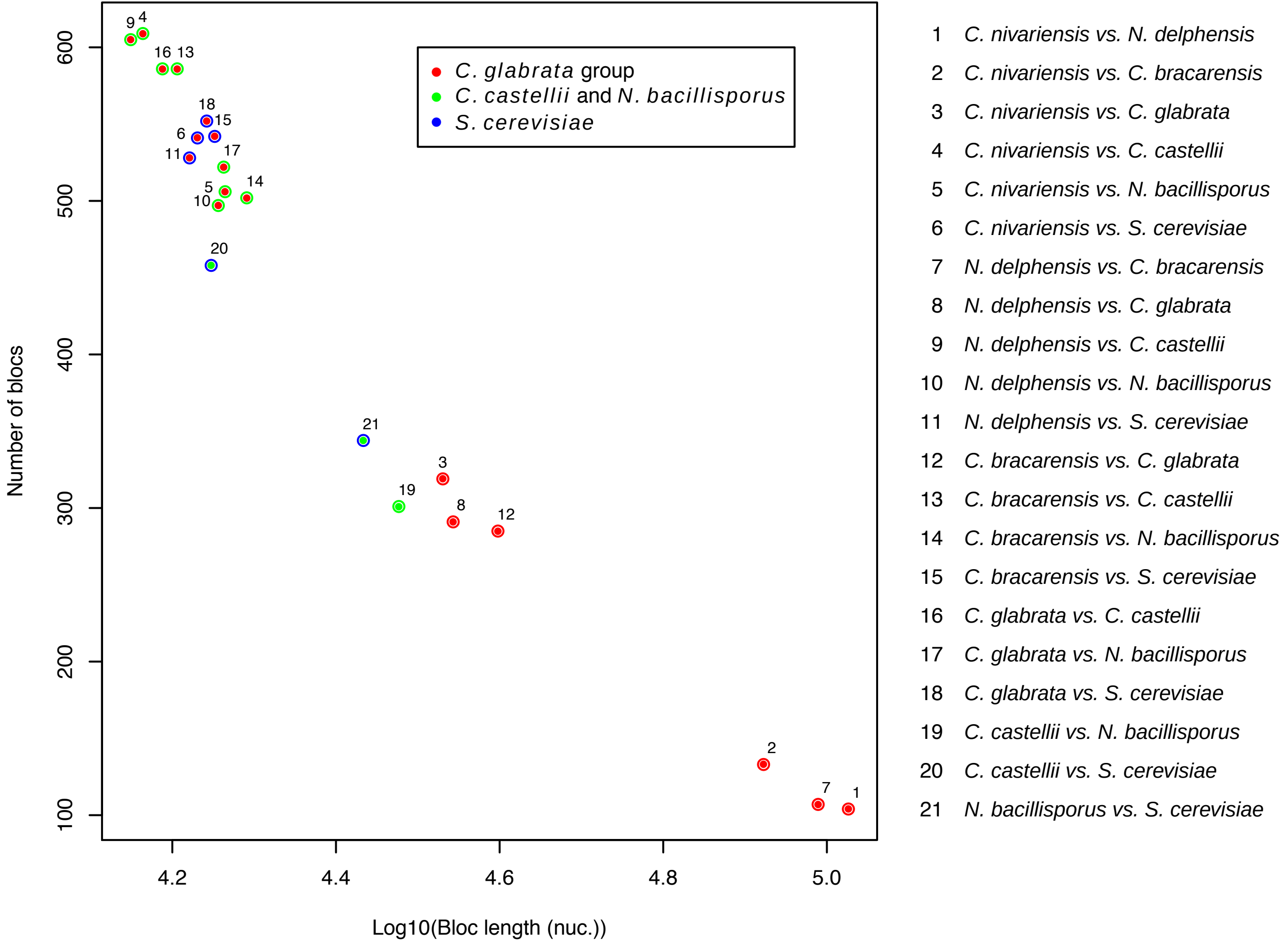
C. glabrata group
C. castellii and N. bacillisporus
S. cerevisiae
Number of blocs
Log10(Bloc length (nuc.))
100
200
300
400
500
600
4.2
4.4
4.6
4.8
5.0
1 C. nivariensis vs. N. delphensis
2 C. nivariensis vs. C. bracarensis
3 C. nivariensis vs. C. glabrata
4 C. nivariensis vs. C. castellii
5 C. nivariensis vs. N. bacillisporus
6 C. nivariensis vs. S. cerevisiae
7 N. delphensis vs. C. bracarensis
8 N. delphensis vs. C. glabrata
9 N. delphensis vs. C. castellii
10 N. delphensis vs. N. bacillisporus
11 N. delphensis vs. S. cerevisiae
12 C. bracarensis vs. C. glabrata
13 C. bracarensis vs. C. castellii
14 C. bracarensis vs. N. bacillisporus
15 C. bracarensis vs. S. cerevisiae
16 C. glabrata vs. C. castellii
17 C. glabrata vs. N. bacillisporus
18 C. glabrata vs. S. cerevisiae
19 C. castellii vs. N. bacillisporus
20 C. castellii vs. S. cerevisiae
21 N. bacillisporus vs. S. cerevisiae
