# Supplementary material for: Comparative genomics of emerging pathogens in the Candida glabrata clade
Source: BMC Genomics. 2013 Sep 14;14:623. doi: 10.1186/1471-2164-14-623 (PMC3847288; doi:10.1186/1471-2164-14-623)
Supplement: Additional file 7 — Histograms of Nakaseomyces genes according to the length of encoded proteins. [file 1471-2164-14-623-S7.pdf]

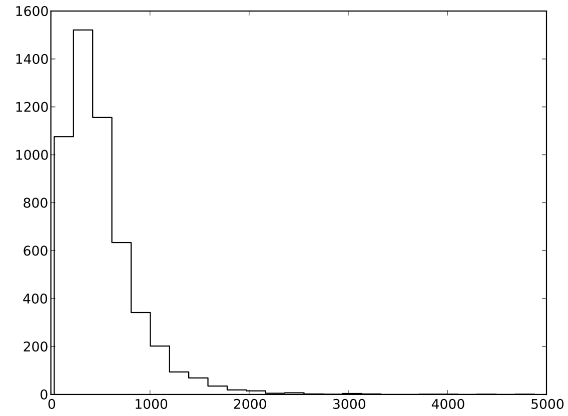

Candida glabrata

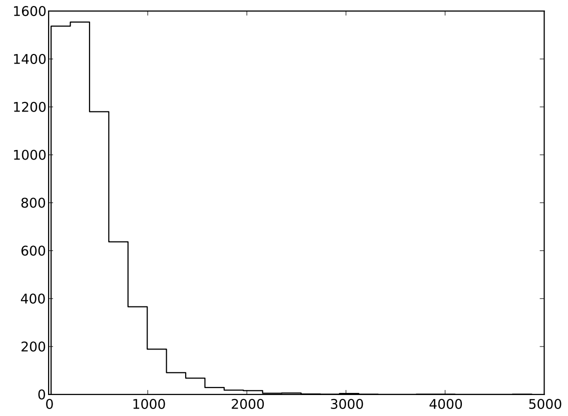

Candida nivariensis

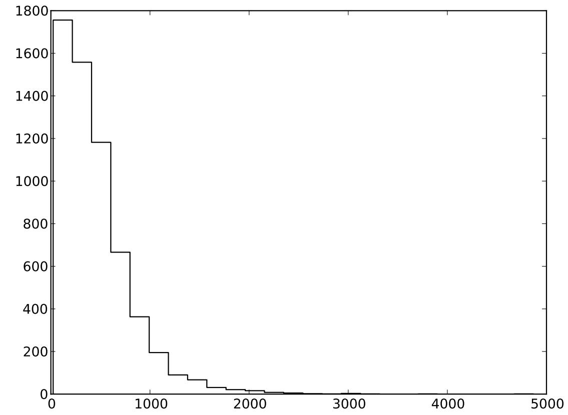

Candida bracarensis

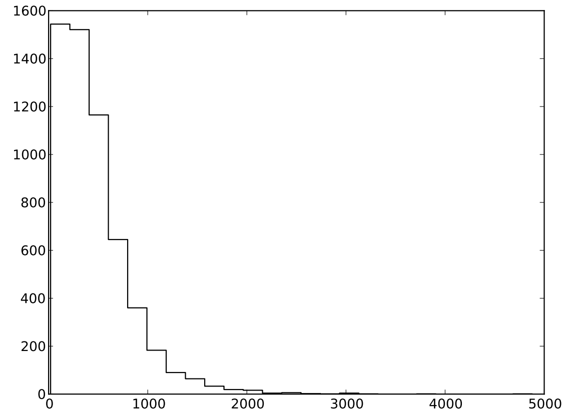

Nakaseomyces delphensis

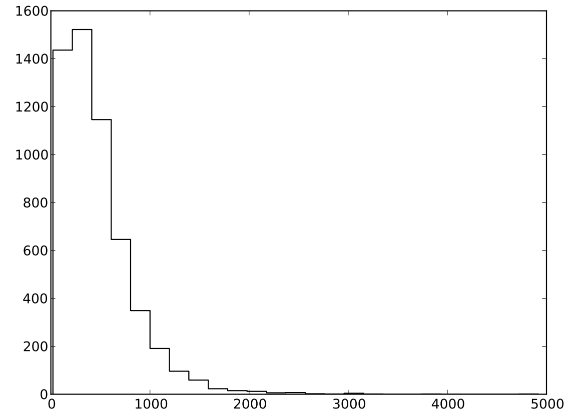

Nakaseomyces bacillisporus

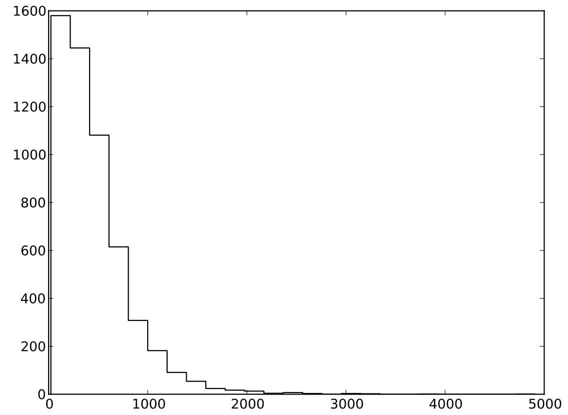

Candida castellii

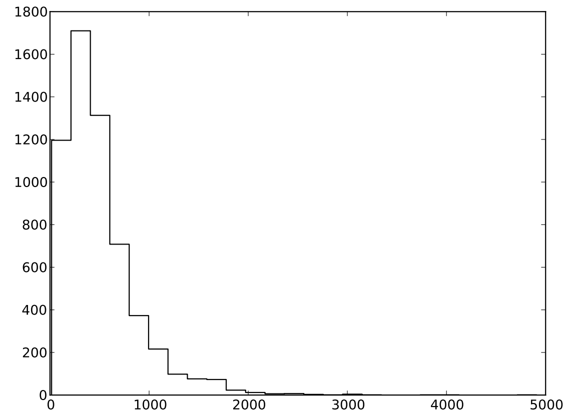

Saccharomyces cerevisiae
