# Supplementary material for: Comparative genomics of emerging pathogens in the Candida glabrata clade
Source: BMC Genomics. 2013 Sep 14;14:623. doi: 10.1186/1471-2164-14-623 (PMC3847288; doi:10.1186/1471-2164-14-623)
Supplement: Additional file 12 — Scenarios for positive selection tests. [file 1471-2164-14-623-S12.pdf]

# LRT A

7 taxa

## Null model

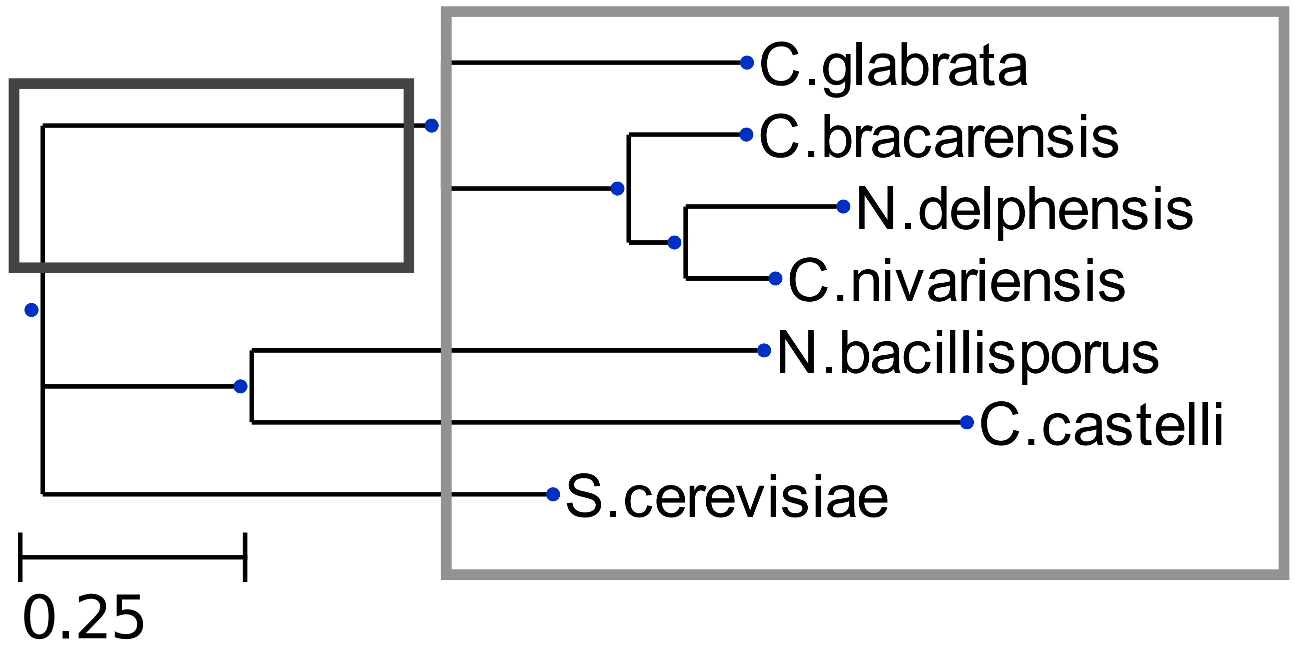


2-rates

## Alternative model

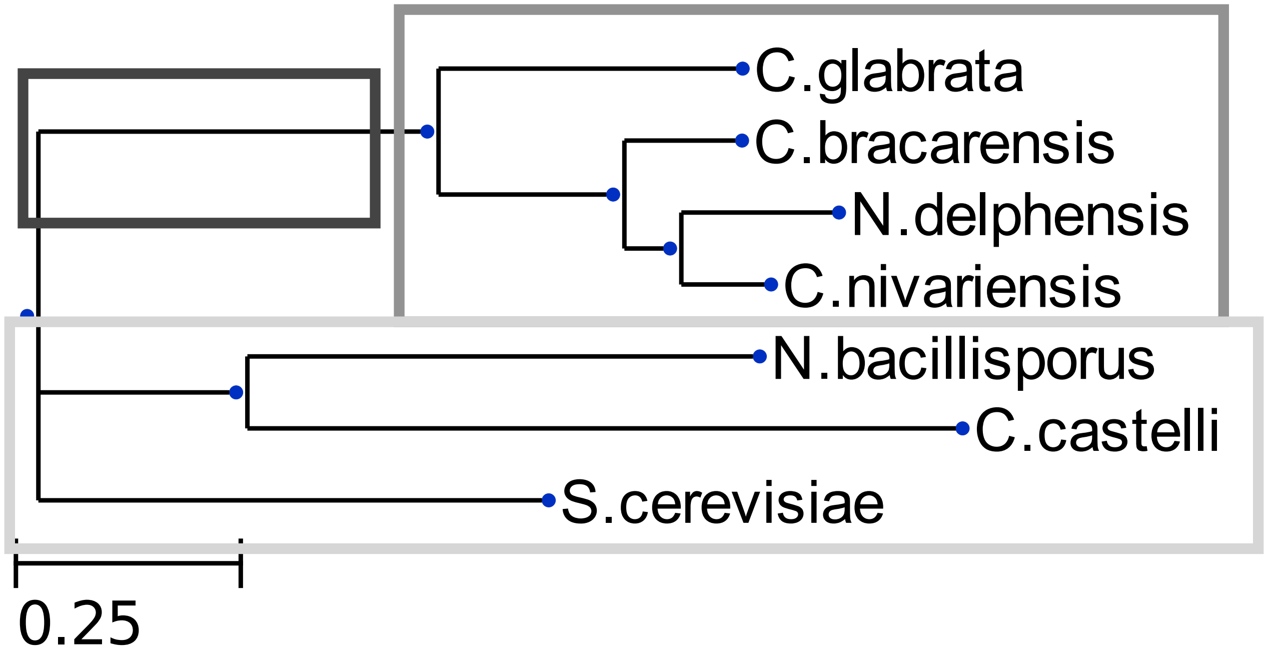


3-rates

# LRT B

5 taxa

## Null model

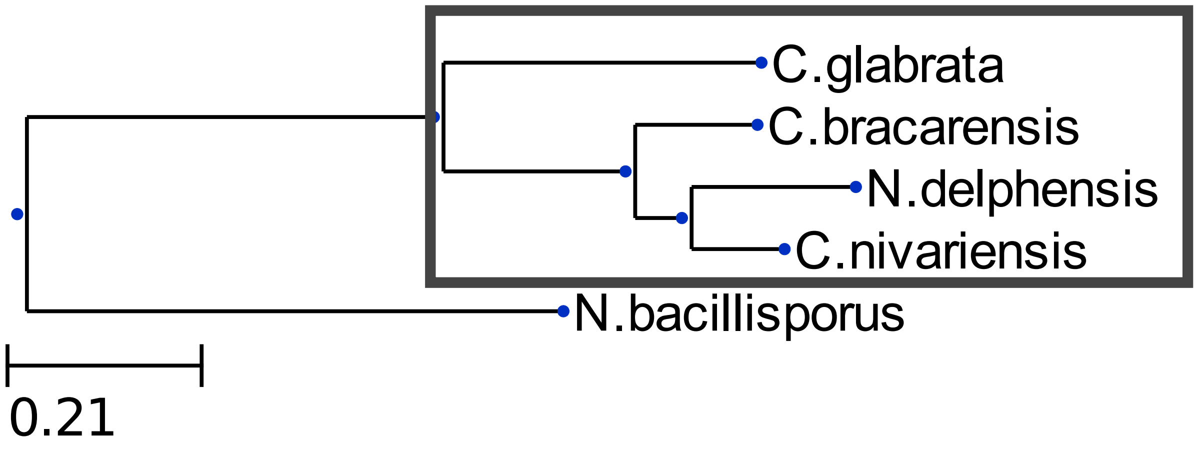


2-rates

## Alternative model

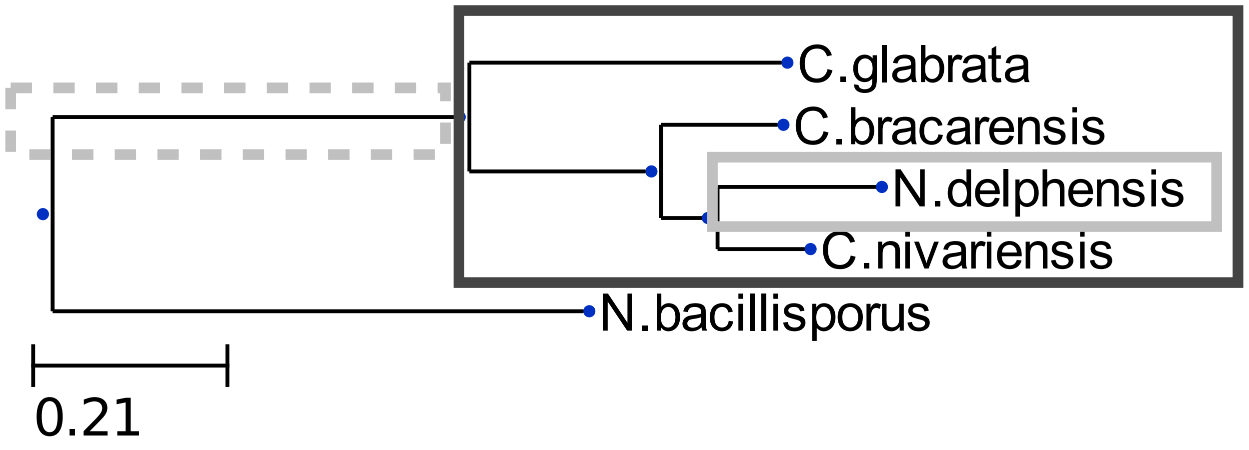


4-rates

# LRT C

5 taxa

## Null model

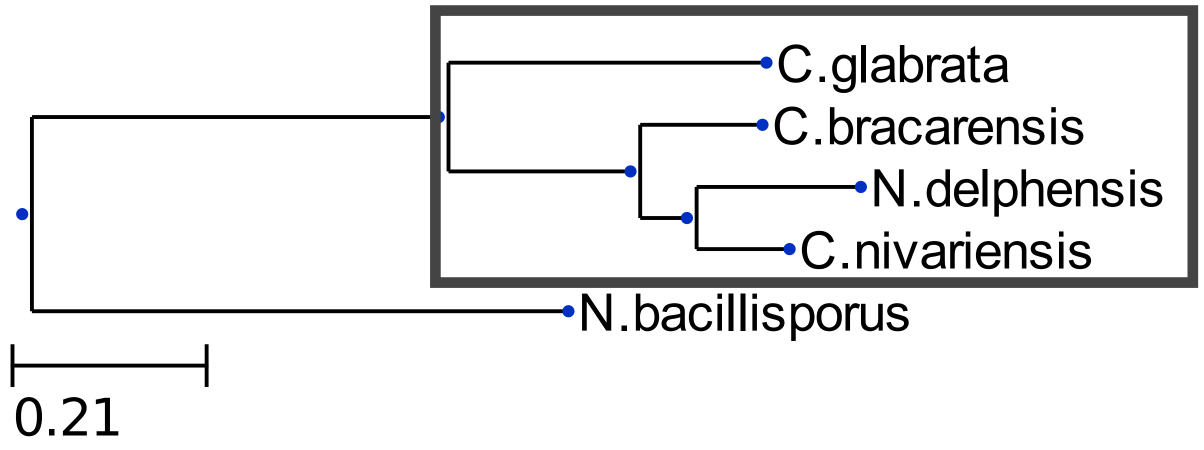


2-rates

## Alternative model

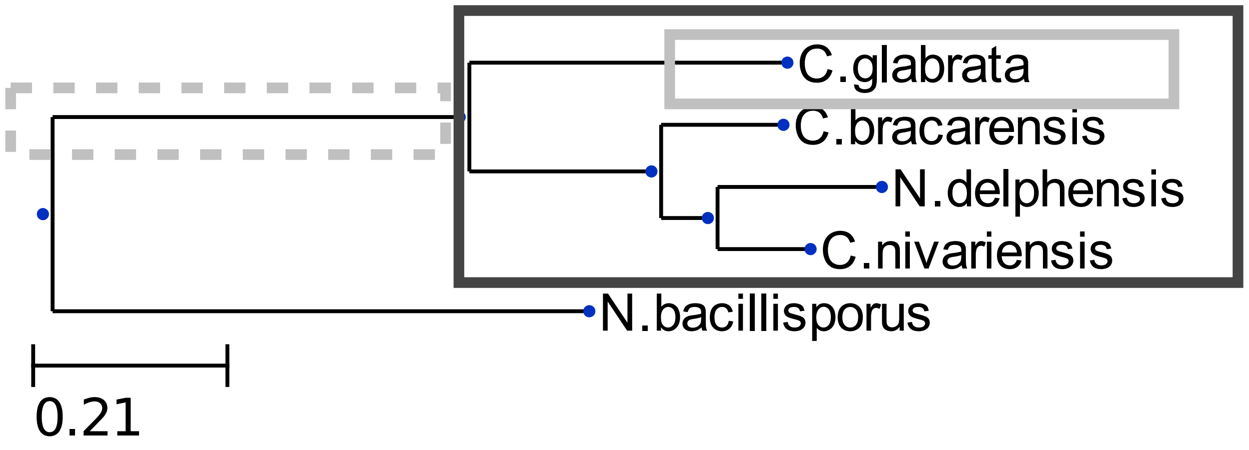


4-rates
