# Supplementary material for: Comparative genomics of emerging pathogens in the Candida glabrata clade
Source: BMC Genomics. 2013 Sep 14;14:623. doi: 10.1186/1471-2164-14-623 (PMC3847288; doi:10.1186/1471-2164-14-623)
Supplement: Additional file 6 — Alignment of putative Ho proteins. “LAGLIDADG” motifs and Nuclear Localization Signals are boxed. [file 1471-2164-14-623-S6.pdf]

```
  N.delphensis  MFDINTTVVMANGEIKKVCELTVNSLVMCDDGTPARITHISSAHHTTYEIYQKTKHRANE
 C. nivariensis MFEINTTVIMANGQIKKVSELTINSLIMCEDGTPARVTKLSKAHHTIYEIFQKTKHRANE
 C. bracarensis MFEKNTTVIMSSGEVQKVSELRVNSLIMCEDGTSARITGLSKARHTVYEIVQKTKHRANE
    C. glabrata MFEKGTFILMADGHLEDISAIKSNSYVMCEDGTPGRVAYTTKAKQTIYEIVQKTKHRANE
  S. cerevisiae MLSENTTILMANGEIKDIANVTANSYVMCADGSAARVINVTQGYQKIYNIQQKTKHRAFE
   C. castellii MLEEKTQVITSHKEIKDVSDLKVNDNLMCDDGTSARIIEIFEDVQPVYEIYQKTKHRANT
N. bacillisporus MLEENTQIITADHRLVEISDLKINDYILCDDGTSAQVTQITRDAQVLYEIKHRTKHRANE

  N.delphensis  GEPGRLDPRRKTVYQRLGFNCTGSHLIPLRVPAIACLENSTIK-PNLTVRWRCLEDVVTN
 C. nivariensis GEPGRLDPRRKTIYQRLGFRCTGSHLLPLRAPAVPSLENSPKK-SNLTVRWRCLQEIITT
 C. bracarensis GEPGRLDPLRKTVYQRLGFCCTGSHVIVLRAPTIPCLENSNKK-SNLTVKWRCLEEVLTH
    C. glabrata GEPGRLDPRRRTVYNRLGFNCSATHKLVLKTPSIPTLENNPRR-PNLTVKWRCLEEILTT
  S. cerevisiae GEPGRLDPRRRTVYQRLALQCTAGHKLSVRVPTKPLLEKSGRNATKYKVRWRNLQQCQTL
   C. castellii GEPGRRDPLRKKIYQRLVLECGAAHKIRLRTSAIPQLVTNSKK-DNCTVCWKDMRQMVTQ
N. bacillisporus QLPGRLDPKRREVYQRLTLNCTAGHKLHLRTPACATIDNSPKK-SNICVRWRDLQEIETS

  N.delphensis  DGRLISIPKNHHKNFPKTQEGFLLADNFIKERSAITGDYIDYLIEVRDLDYLDTSMRVTS
 C. nivariensis DGRSISIPKNHHKNFPKTEEGYCQAQNFITEKRVINGDYIDFKIEVRDFDYLDASMRVTS
 C. bracarensis DGRPISIPKNHHKVFPKTELGYIQAQSFINEKRSINGDYLTYEIQVRDLDYLDASMRVTS
    C. glabrata DGRAITVPKNHHKNFPKTKEGQLQAQNFMRDKRLIWGPDIDYEIQVRDLEYLDASMRVTS
  S. cerevisiae DGRIIIIPKNHHKTFPMTVEGEFAAKRFIEEMERSKGEYFNFDIEVRDLDYLDAQLRISS
   C. castellii DGRILDVPKIHHKNFPKSSEGIQAAQYFMSGITSQMGSYLYYDMQVMDLEYLDPHVRVTS
N. bacillisporus DQRKIIVPKTHHKNFPKTNEGIQAAELFATDKIELNGAFLEYDIQVIDLDYLDAQIRVSS

  N.delphensis  ALKCSPILCGNGILSKFLSGKPHLITPSITAMAWLLGLWIGDGTTKEPEITMDSLDAPLM
 C. nivariensis ALMCSPILVGNGVLSKFLSGQPHLVTPSITVMAWLLGLWIGDGTTKEPEITVDSHDVSLM
 C. bracarensis TMKCNPILVGNGILSHHLSGQRHLITPAITAMAWLLGLWIGDGTTKEPEITVDSQDIDLM
    C. glabrata TLKCNPVFSGNGILSNFLSGQRHLITPAIVSMAWLLGLWIGDGTTKEPEITVDSVDEKLM
  S. cerevisiae CIRFGPVLTGNGVLSKFLTGRSDLVTPAVKSMAWMLGLWLGDGTTKEPEISVDSLDPKLM
   C. castellii YMEFHPVTKGNGIISKHLTGQKGLITPKVLGMAWMLGLWIGDGTTKEPEISMDSSDTELI
N. bacillisporus NMKFCPIMGGNGILANFLTGQKEIMTPSVICMAWMLGLWIGDGTTKEPEISVDSSDVELM

  N.delphensis  NSLIVLGRKWGIYPTYKDEKVPLRAKHVRLYYGNEPEEKRKTRNLRKNNPFWNTVLALGF
 C. nivariensis NSLIVMGRKWGIYPTYKDERVPLRAKHVRLYYGNEPNEKRKTRNLRKNNPFWNTVLSLGF
 C. bracarensis NSLTVLGRKWGIYPTYKDEKIPLRAKHVRLYYGNEIEEKRKTRNLRKNNPFWNTLLTLGF
    C. glabrata ESLTVLGRYWGLYPTYKDEKVPLRAKHVRLYYGKGPEERRKTRNLRKNNPFWNTIQNLGI
  S. cerevisiae ESLRENAKIWGLYLTVCDDHVPLRAKHVRLHYGDGPDENRKTRNLRKNNPFWKAVTILKF
   C. castellii AGLIEISSQWGIFPAFKDEFVPLRAKHVRLYYGNGLDENRKTRQLRKNNPFWNTITSLKF
N. bacillisporus RGLTELAEQWGIYPYYKDELVPLRAKHVRLYYGKEPSENRKTRNLRKNNPFWNAVTKLKF

  N.delphensis  KKENSGEKYVPEFMWTEDIEIREAFLAGLIDSDGYVSKRKDNPD-VYKVSIQTIYPCIMD
 C. nivariensis KKAISGEKYVPEFMWTEDIEVREAFLAGLIDSDGYVVKRKENPD-TYKVSIQTVYPCIMD
 C. bracarensis KKETSGEKQVPSFMWTEDIEVREAFLAGLIDSDGYVVKRKENPN-TYKVSIQTVYSSIMD
    C. glabrata KREIDGEKQVPEFMWHEDIEIREAFLAGLIDSDGYVVKRKENPD-VYKVSIQTIYPSVMN
  S. cerevisiae KRDLDGEKQIPEFMYGEHIEVREAFLAGLIDSDGYVVKKGEGPE-SYKIAIQTVYSSIMD
   C. castellii KRDTDGVKDVPEFLWNEDVEIREAFLAGLIDSDGYVVTRNSSSTPQFKVSIQTVYSTVMN
N. bacillisporus KRDLDGVKQVPEFMWTEQLEVREAFLAGLIDSDGYVLNNGEN----YKVSIQTVYASVMN

  N.delphensis  AVVHIARSLGISATVTTRSARPEVIEGRLVNCQFTYDCNISGSSALQNVLSYCRSGHKRR
 C. nivariensis AIVNIARSLGISATVTTRSARPEVIEGRLVNCQFTFDCNISGSSALQNVLSYCRSGHKRR
 C. bracarensis AIVHIARSLGISATVTTRSARPEVIEGRLVSCQFTYDCNISGASALQNILSYCRSGHKRQ
    C. glabrata AIVHIARSLGISATVTTRSARNEVIEGRRVQCRFTYDCNISGSTPLQNVLSYCRSGHKRK
  S. cerevisiae GIVHISRSLGMSATVTTRSAREEIIEGRKVQCQFTYDCNVAGGTTLQNVLSYCRSGHKTR
   C. castellii AVAHIARSLGISVTITTRSARSEKIEDRTVQCRFTYDCCISGGAPLQKVLSLCHSKTKRR
N. bacillisporus AIAHISRSLGLSVTVTTRSARTEKIEGRTVQCRFTYDCFISGGLALQNVLSYCRSTFKRR

  N.delphensis  KAPESVIREPQFFGFIDKKISEADVKGIHFEDERNILLGNKVVAHCCKQECLTEGNRLLD
 C. nivariensis CPPEVVIREPQFVGFIDQKKSESEVIGIHLDKQQNIILGNKLIAHTCGPECLTEGNRQFD
 C. bracarensis DPPEVVLRDPQFVGFIDQKISELEVVGIHLQTPKNILLANKIVARSCGKECLIEGNRHMD
    C. glabrata PAPEVVIRDPQYVGFIDRKLREAEVYGIHLDQPRNILLGNKIVVYSCTECCETDAVHITK
  S. cerevisiae EVPPIIKREPVYFSFTDDFQGESTVYGLTIEGHKNFLLGNKIEVKSCRGCCVGEQHKISQ
   C. castellii PDPGPFIRKPVYFGFDVKHKGPSKMVGIKLDASKPIMLANKMVSLPCTEACKVDHDIPYV
N. bacillisporus PKPTNFDRQPVYFGFDVERKGPASVIGFKTDSKKAVLLGNKVSLPICNDQCTKDYDIPYV

  N.delphensis  TKKLKYCVSCPRSGVRYFYRDWTGKNRVCGRCYGRYKFSGYRCVNCMYVPEAREVKKAKM
 C. nivariensis TKNLKYCISCPRSGVRYFYRDWSGTNRICGRCYGRYKFSGYRCVNCKYIPEAREIKKAKM
 C. bracarensis IRNLKYCISCPRTGVRYFYRDWTGKNRICSRCYGRYKFSGYRCLNCKYIPEAREVKKAML
    C. glabrata IKNLKHCVSCPRTGVRYFYRDWTGKSHVCGRCYGRYKFSGYRCLHCKYIPEAREIKKARM
  S. cerevisiae KKNLKHCVACPRKGIKYFYKDWSGKNRVCARCYGRYKFSGHHCINCKYVPEAREVKKAKD
   C. castellii NPRIKQCIACPRRGVRYFYKDWTGESSVCGRCYARYKFSGHRCLNCKYVPDSREVKKARM
N. bacillisporus NKKLKQCISCPRRGIRYFYKDWTGTGRVCGRCYGRYKFSGHRCLNCKYVPEAREVKKARS

  N.delphensis  KGEELGIDPTGIPVRGICCPRCSGILNFDEIRGPSSAHRRAMIN----------------
 C. nivariensis KGEELGIDPSGIPVRGICCPRCCGILLYDEVRGPSANIRRVMLN----------------
 C. bracarensis KGEQLGIDSTGFPVRGCCCPRCSGIMKYDEVRGPCTITRHAMLN----------------
    C. glabrata RGEETRLFEQSY-ITGLVCSRCEGILTFDEIRGPAKKQAVQ-------------------
  S. cerevisiae KGEKLGITPEGLPVKGPECIKCGGILQFDAVRGPHKSCGNNAGARIC-------------
   C. castellii LGEKVGITPDGVMTQGYPCSSCGGILIFDAIRGPRKESRIALEAC---------------
N. bacillisporus QGTKIGFNEHGNAVQGYPCVSCGGVLVYDEIRGPRKTAVAPPSPPTSPPTSPPTSPPTSP

  N.delphensis  ---------------------------------------------------------
 C. nivariensis ---------------------------------------------------------
 C. bracarensis ---------------------------------------------------------
    C. glabrata ---------------------------------------------------------
  S. cerevisiae ---------------------------------------------------------
   C. castellii ---------------------------------------------------------
N. bacillisporus SSPSVSPSPSSTPSPSPPPSPFRSFTPSPSFPPTSPPSSPPPFSIESIPVTDSSVYI
```
